# Phytochemical Profiles and Antimicrobial Activity of Selected *Populus* spp. Bud Extracts

**DOI:** 10.3390/molecules29020437

**Published:** 2024-01-16

**Authors:** Piotr Okińczyc, Jarosław Widelski, Kinga Nowak, Sylwia Radwan, Maciej Włodarczyk, Piotr Marek Kuś, Katarzyna Susniak, Izabela Korona-Głowniak

**Affiliations:** 1 Department of Pharmacognosy and Herbal Medicines, Faculty of Pharmacy, Wroclaw Medical University, Borowska 211a, PL-50-556 Wrocław, Poland; maciej.wlodarczyk@umw.edu.pl (M.W.); piotr.kus@umw.edu.pl (P.M.K.); 2 Department of Pharmacognosy with Medicinal Plants Garden, Medical University of Lublin, Chodźki 1, PL-20-093 Lublin, Poland; 3 Institute of Dendrology, Polish Academy of Sciences, Parkowa 5, PL-62-035 Kórnik, Poland; knd@man.poznan.pl; 4 Laboratory of Elemental Analysis and Structural Research, Faculty of Pharmacy, Wroclaw Medical University, Borowska 211a, PL-50-556 Wrocław, Poland; sylwia.radwan@umw.edu.pl; 5 Department of Pharmaceutical Microbiology, Medical University of Lublin, Chodźki 1, PL-20-093 Lublin, Poland; katarzyna.susniak@umlub.pl (K.S.); iza.glowniak@umlub.pl (I.K.-G.)

**Keywords:** poplar, *Populus nigra*, *Populus trichocarpa*, *Populus tacamahaca*, UHPLC, LC-MS, qTOF-MS/MS, MS, antimicrobial activity, *Helicobacter pylori*, chemical profile

## Abstract

Buds of poplar trees (*Populus* species) are often covered with sticky, usually polyphenol-rich, exudates. Moreover, accessible data showed that some *Populus* bud extracts may be excellent antibacterial agents, especially against Gram-positive bacteria. Due to the fragmentary nature of the data found, we conducted a systematic screening study. The antimicrobial activity of two extract types (semi-polar—ethanolic and polar—ethanolic-water (50/50; *V*/*V*)) from 27 bud samples of different poplar taxons were compared. Antimicrobial assays were performed against Gram-positive (five strains) and Gram-negative (six strains) bacteria as well as fungi (three strains) and covered the determination of minimal inhibitory, bactericidal, and fungicidal concentrations. The composition of extracts was later investigated by ultra-high-performance liquid chromatography coupled with ultraviolet detection (UHPLC-DAD) and with electrospray-quadrupole-time-of-flight tandem mass spectrometry (UHPLC-ESI-qTOF-MS). As a result, most of the extracts exhibited good (MIC ≤ 62.5 µg/mL) or moderate (62.5 < MIC ≤ 500 µg/mL) activity against Gram-positives and *Helicobacter pylori*, as well as fungi. The most active were ethanolic extracts from *P*. *trichocarpa*, *P*. *trichocarpa* clone ‘Robusta’, and *P*. *tacamahaca* × *P*. *trichocarpa*. The strongest activity was observed for *P*. *tacamahaca* × *P*. *trichocarpa*. Antibacterial activity was supposedly connected with the abundant presence of flavonoids (pinobanksin, pinobanksin 3-acetate, chrysin, pinocembrin, galangin, isosakuranetin dihydrochalcone, pinocembrin dihydrochalcone, and 2′,6′-dihydroxy-4′-methoxydihydrochalcone), hydroxycinnamic acids monoesters (*p*-methoxycinnamic acid cinnamyl ester, caffeic acid phenethylate and different isomers of prenyl esters), and some minor components (balsacones).

## 1. Introduction

Poplars are high trees that belong to the genus *Populus* L. of Salicaceae Mirb. family due to the traditional organism systematics of Carolus Linnaeus [1,2], while the (*Salix* + *Populus*) clade is also classified into a higher clade of ((Goupiaceae + Violaceae) (Passifloraceae (Lacistemataceae + Salicaceae))) by Angiosperm Phylogeny Group [3]. Different authors distinguished 6–7 sections of the Populus genus, but this classification is evolving [2]. The deeper systematics of the genus *Populus* is complex and still discussed due to many factors. It is claimed that *Populus* specimens are difficult to identify accurately. Moreover, most poplars are known for their ease of spreading and crossbreeding [1,2,4]. A typical example is American *P. balsamifera* L., which quickly spread in Europe and produced intersectional hybrids with Europe-native *P*. *nigra* L. (black poplar) [4]. As a result, pure specimens of black poplars are relatively rare today [1,4]. Another issue is differences in statements of species classifications. According to World Flora Online [5], traditionally distinguished species like *P. maximowiczii* Henry, *P. suaveolens* Fisch. ex Loudon, and *P. koreana* Rehder are nowadays recognized as synonyms of *P. suaveolens* Fisch. A similar situation occurs for *P. balsamifera* and *P*. *trichocarpa* [2]. In summary, different descriptions of *Populus* species exist in the literature. For this reason, the system of *Populus* species classification adopted for this work should be defined. In the present manuscript, a traditional division of species described by Bugała in his monograph elaboration and further modified by Korbik [1,2] was used. This decision was made due to the widespread use of traditional names in the literature, even in 2023.

Apart from systematics, the Salicaceae family is known for famous medicinal plants, especially willows (*Salix* genus). Willow bark contains biologically active compounds, especially salicylate-like phenolic glycosides [6]. This group of metabolites includes glycosides and glycoside-esters (e.g., salicin and salicortin), aromatic acids (cinnamic and hydroxycinnamic acids), and others. It is well known that salicylates exhibit anti-inflammatory activity [6]. Raw herbal materials from *Salix* species, especially bark, rich in salicylate-like phenolic glycosides, are still used in cold and mild rheumatic diseases. Moreover, the bark of willow species has its monograph (*Salicis cortex*) in many official pharmacopeias [7,8].

In the case of the poplars (*Populus* genus), their organs (bark [9], leaves [10], buds [11,12]) also contain anti-inflammatory salicylate-like phenolic glycosides. For this reason, poplar’s organs are also used in folk medicine to treat gout [13]. However, unlike willows, poplars are not widely included in pharmacopeias. Leaves of poplars (*Populi folium*) are included in the national part of Polish Pharmacopeia [14]. In case of buds, some species such as *P. nigra* L., *P*. *balsamifera* L., *P*. *canadensis* Marsh., *P*. *laurifolia* Ledeb., and *P*. *suaveolens* Fisch. are plant sources of *Populi gemmae* in the Russian Pharmacopeia [15]. However, the *Populi gemmae* monograph is absent in European Pharmacopoeia 11 and USP-NF 2023.

Apart from salicylate-like phenolic glycosides, buds of many *Populus* species are covered by sticky, resinous exudates [1,16], an additional source of biologically active components. The amounts, seasons, and periods of resin production depend on species and environmental factors. Buds of some species (e.g., *P. tremula* L.) are only resinous for a short period before cracking in spring, while others are sticky almost all year [1]. The composition of *Populus* resins is very complex but specific for species. For these reasons, a comparative analysis of bud exudates composition may be helpful for chemotaxonomic purposes [16]. At this point, the main components of *Populus* resinous exudates were defined as phenols, volatile and non-volatile terpenes and terpenoids, and other substances [16,17].

Biologically, resins form a protective layer on buds, making them less sensitive to wetness, cold, and attacks of pathogenic microorganisms and parasites and less attractive for herbivores. On the one hand, poplar resins often contain relatively high concentrations of polar free phenolic acids; however, more apolar components such as flavonoid aglycones and phenolic acids esters are also detected [16,17]. Surprisingly, one of the rarest substances in resins are glycosides, e.g., salicylate-like glycosides. Literature data have reported their presence in extracts from whole buds [11,12,18] but did not focus unambiguously on resins. Thus, one can guess that salicylates may be components of buds’ interior green tissues, not bud exudates.

Resins of the *Populus* genus are plant precursors of a bee product known as propolis (or bee glue). It is well-known for multiple medicinal activities such as wound treatment, anti-inflammatory, antioxidant, and antimicrobial. These properties were also reported for poplar bud’ extracts [11,19,20,21]. Researchers reported differences between propolis and poplar buds; however, it is impossible to claim if propolis or poplar buds have more potent medicinal properties. Moreover, *Apis mellifera* L. bees do not use resins from all available *Populus* species to produce propolis. It was observed that their preferences for local species may be so strong that foreign poplar trees are ignored. Sometimes, non-poplar species (e.g., birch) are preferred over foreign *Populus* specimens [22]. The factors impacting bees’ decisions remain unknown. It is suspected that components of some *Populus* species resins may be toxic or repellent to bees. That is why some poplars may contain highly active components not observed in propolis research. Moreover, literature data on *Populus* buds are limited compared to propolis or poplar leaves research. So far, phytochemical analyses for species other than *P*. *nigra, P. balsamifera*, and *P. tremula* have focused mainly on GC-MS profiling [23,24,25,26,27,28,29,30,31,32], while LC-MS and LC-DAD investigations are more limited [16,18,28,29]. Moreover, the chemical composition of buds is not yet defined for every *Populus* species.

Our manuscript compares phytochemistry with antimicrobial properties of ethanol and ethanol/water (50/50; *V*/*V*) extracts of poplar buds. *Populus* species were selected due to the high production of resins (the viscosity of buds before cracking was evaluated in preliminary research) and the expected activity. Section 3 (Materials and Methods) contains a complete list of investigated poplar species. Instrumental analysis was performed using LC-UV-ESI-QTOF-MS/MS due to the expected high amounts of polyphenols and only a few similar works. Solvents used in extraction were chosen due to desired components and previous optimization. Ethanol dissolves buds’ resins and their less polar components, such as flavonoid aglycones and hydroxycinnamic acids esters. More polar constituents (e.g., salicylate-like glycosides) from buds’ green tissues were extracted by ethanol with water (50:50, *V*/*V*). Antimicrobial activity screening against bacterial (Gram-positive and Gram-negative) and fungal strains was based on previous experience and the expected activity of propolis [33], as well as results for *P*. *nigra* and *P*. *tremula* buds [20]. To our knowledge, LC-MS-UV-ESI-qTOF-MS/MS analysis and antimicrobial screening were performed for the first time for poplar bud extracts of most *Populus* species, excluding *P. nigra*, *P. balsamifera,* and *P. tremula*. Moreover, the activity of all extracts against *Helicobacter pylori* was also tested for the first time.

## 2. Results and Discussion

Poplar buds, their resins, and propolis are similar, but they are not the same type of plant material and thus should not be replaced by each other. The *Populus* buds’ composition and activity data are relatively sparse, particularly regarding propolis. Therefore, the extensive comparative studies of the phytochemistry and biological activity of poplar buds conducted in this study constitute a valuable contribution to this field.

### 2.1. LC-UV-ESI-qTOF-MS/MS Profile of Extracts

**Figure 1:**
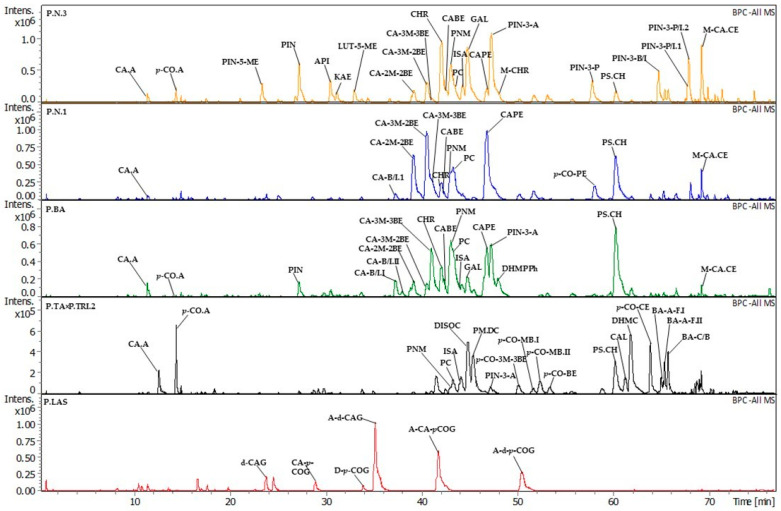
LC-MS chromatograms of *Populus* buds EtOH extracts representing five chemical groups (negative mode, base peak chromatograms). Figure legend: Lanes: orange—**P.N.3**—*P. nigra*, sample 3 (flavonoid type); blue—**P.N.1**—*P*. *nigra*, sample 1 (hydroxycinnamic monoesters type); green—**P.BA**—*P*. *balsamifera* (hydroxycinnamic monoesters + flavonoid type); black—**P.TA** × **P.TRI.2**—*P*. *tacamahaca* × *P*.*trichocarpa*, sample 2 (mixed type); red—**P.LAS**—*P*. *lasiocarpa* (hydrocinnamic acids glycerides type). Component abbreviations: **A-CA-*p*-COG**—2-Acetyl-1-caffeoyl-3-*p*-coumaroylglycerol; **A-d-CAG**—2-Acetyl-1,3-di-caffeoylglycerol; **A-d-*p*-COG**—2-Acetyl-1,3-di-*p*-coumaroylglycerol; **API**—apigenin; **BA-A-F.1**—Balsacone A/B/E/F isomer I; **BA-A-F.2**—Balsacone A/B/E/F isomer II; **BA-C/B**—Balsacone C or D; **CA.A**—Caffeic acid; **CA-2M-2BE**—Caffeic acid 2-methyl-2-butenyl ester; **CA-3M-2BE**—Caffeic acid 3-methyl-2-butenyl ester; **CA-3M-3BE**—Caffeic acid 3-methyl-3-butenyl ester; **CA-B/I.1**—Caffeic acid butyl or isobutyl ester isomer I; **CA-B/I.2**—Caffeic acid butyl or isobutyl ester isomer II; **CABE**—Caffeic acid benzyl ester; **CAL**—2′,6′-Dihydroxy-4′,4-dimethoxydihydrochalcone (calomelanone); **CA-*p*COG**—Caffeoyl-*p*-coumaroylglycerol; **CAPE**—Caffeic acid benzyl ester; **CHR**—Chrysin; **d-CAG**—di-Caffeoylglycerol; **DHMC**—2′,6′-Dihydroxy-4′-methoxydihydrochalcone; **DHMPPh**—2′,6′-Dihydroxy-4′-methoxypentanophenone; **DISOC**—Isosakuranetin dihydrochalcone; **d-*p*-COG**—1,3-di-*p*-Coumaroylglycerol; **GAL**—Galangin; **ISA**—Isosakuranetin; **KAE**—Kaempferol; **LUT-5-ME**—Luteolin 5-methyl ether; **M-CA.CE**—Metoxycinnamic acid cinnamyl ester; **M-CHR**—Methoxychrysin; **P.C**—Pinocembrin chalcone; ***p*-CO.A**—*p*-Coumaric acid; ***p*-CO-3M-3BE**—*p*-Coumaric acid 3-methyl-3-butenyl ester; ***p*-CO-BE**—*p*-Coumaric acid benzyl ester; ***p*-CO-CE**—*p*-Coumaric acid cinnamyl ester; ***p*-CO-MB.I**—*p*-Coumaric acid 3-methyl-2-butenyl or 2-methyl-2-butenyl ester isomer I; ***p*-CO-MB.II**—*p*-Coumaric acid 3-methyl-2-butenyl or 2-methyl-2-butenyl isomer II; ***p*-CO-PE**—*p*-Coumaric acid phenethyl ester; **PIN**—Pinobanksin; **PIN-3-A**—Pinobanksin 3-acetate; **PIN-3-P**—Pinobanksin 3-propanoate; **PIN-3-B/I**—Pinobanksin 3-butanoate or -isobutanoate; **PIN-3-P/I.1**—Pinobanksin 3-pentanoate or -isopentanoate isomer I; **PIN-3-P/I.2**—Pinobanksin 3-pentanoate or -isopentanoate isomer II; **PIN-5-ME**—Pinobanksin 5-methyl ether; **PM.DC**—Pinocembrin dihydrochalcone; **PNM**—Pinocembrin; **PS.CH**—Pinostrobin chalcone.

Complete results of LC-UV-ESI-qTOF-MS/MS are presented in Table 1 (identification of components in *Populus* bud extracts by LC-UV-ESI-qTOF-MS/MS) and in the supplement (Table S1. Relative abundance of extracts components and buds’ extraction yield). A selection of chromatograms is given in Figure 1 (LC-MS chromatograms of *Populus* bud EtOH extracts represent five different chemical groups). The identification of components was based on retention times of chromatographic peaks and UV spectra, and calculated formulas of deprotonated molecular ions as well as MS/MS fragmentation spectra. Due to the different confidence levels, the obtained information was divided into four groups—A, B, C, and D (see Section 3 and Table 1 for details). Confidence levels A and B mean reliable identification, while levels C and D are tentative.

More than 300 unique components were observed in UV and MS chromatograms. Because most of them remained at trace level (MS or UV peak), Table 1 and Supplementary Table S1 were limited to 223 components. Among them, 163 substances were identified (confidence levels A and B) or tentatively identified (confidence levels C and D). The substitution positions of glycerol by hydroxycinnamic acids were proposed by comparison with previous research [33] and literature [17,19,23]. So far, it has been proved that more symmetric hydroxycinnamic acid glycerides dominate over non-symmetric glycerides in GC-MS research [17,19,23]. For example, 2-acetyl-1,3-di-*p*-coumaroyl glycerol had a higher concentration than 3-acetyl-1,2-di-*p*-coumaroyl glycerol in *P*. *tremula* buds [23] and further in propolis [17,19]). Differences in concentration and ionization were used to identify isomers of caffeic acid *p*-coumaric acids methylbutenyl ester [33].

Most of these components were phenols and polyphenols, classified as free hydroxycinnamic acids, salicylate-like phenolic glycosides, hydroxycinnamic acids monoesters, hydroxycinnamic glycerides, other polyphenols, and non-polyphenols. In terms of compound numbers, the richest phenols and polyphenols class was flavonoids (73 components), followed by hydroxycinnamic acids monoesters (35 comp.), others polyphenols (22 comp.), hydroxycinnamic acids glycerides (13 comp.), salicylate-like glycosides (9 comp.), and free phenolic acids (6 comp.), respectively. Only four components were classified as non-polyphenols; most unidentified components were probably also non-polyphenols.

Regarding the components’ relative abundance, poplar bud extracts were mainly rich in flavonoids, hydroxycinnamic acid monoesters, and hydroxycinnamic glycerides. Most observed substances easily produced ions in negative mode; therefore, their relative abundance was calculated from mass chromatograms. In contrast, tectochrysin, pinostrobin, and ferulic acid benzyl ester did not produce ions, or the signals were weak under standard conditions. For those substances, the relative abundance was obtained by comparison of UV_max_ chromatograms in 280 nm.

Chromatographic profiles of ethanolic (EtOH) and water/ethanol (50/50; *V*/*V*) (W/E) extracts exhibited qualitative and quantitative differences. EtOH extracts were more abundant in a number of components than W/E, but some substances were present only in W/E. Most observed compounds remained at an ion trace level, and only unidentified component 2 (RT 0.87 min, [M−H]^−^ at 195.0515 *m*/*z*) exhibited low and average relative abundance. Moreover, the most common components of EtOH and W/E extracts exhibited higher relative abundance in EtOH. Rarely were W/E extracts more abundant in common substances. For example, pinobanksin was more abundant in W/E of P.LA, P.M×P.B, and P.N3, while in EtOH for the rest of the samples. In summary, ethanol turned out to be a more appropriate solvent than water/ethanol (50/50; *V*/*V*) for batch extraction and further chemometric analysis of poplar buds regarding the number of extracted compounds and their relative abundance.

The relative abundance of most components was obtained due to deprotonated pseudomolecular ion intensity in a single chromatographic peak (see details in Section 3). Only pinostrobin and tectochrysin relative amounts were based on UV peak intensity due to their weak ionization in negative mode.

*Populus* bud extracts were divided into phytochemical groups. The division was based on the presence of dominant components. Domination was determined based on deprotonated pseudomolecular ion intensity (see Supplementary Table S1). Because chromatographic analyses of EtOH extracts had stronger and more numerous peaks of components, they were selected to direct the division. As a result, extracts were aggregated into several subtypes: (1) flavonoid, (2) flavonoid + hydroxycinnamic acid monoesters, (3) hydroxycinnamic acid monoesters, (4) hydroxycinnamic acid glycerides, and (5) mixed ones.

A considerable group of six EtOH extracts was of (1) flavonoid type (P.DE—*P*. *deltoides*; P.DE × P.N.—*P*. *deltoides* × *P*. *nigra*; P.ER—*P*. ‘Eridano’ (*P*. *deltoides* × *maximowiczii* clone Eridano); P.LAU—*P*. *laurifolia*; P.MAX—*P*. *maximowiczii*; P.N.3—*P*. *nigra*, sample 3). The flagship compounds in this group included pinobanksin 5-methyl ether, pinobanksin, chrysin, pinocembrin, galangin, and pinobanksin 3-esters (acetate—main—followed by propanoate, butanoate or isobutanoate, and pentanoate or isopentanoate isomer II). Pinocembrin and pinobanksin 3-acetate usually occurred with the strongest signal among these components. The hydroxycinnamic acid monoesters were similar to the previous type but not always present, even if they gave lower peaks (except metoxycinnamic acid cinnamyl ester in P.N.3 and P.ER).

Most EtOH extracts (10/27) were in (2) flavonoid + hydroxycinnamic acid monoesters type (P.BA—*P*. *balsamifera*; P.CA—*P*. *cathayana*; P.KOM—*P*. *komarovii*; P.M×P.B—*P*. *maximowiczii* × *P*. × *berolinensis* (*P*. *laurifolia* × *P*. *nigra* ‘Italica’), P.×PE.1, P.×PE.2—*P*. × *petrovskiana* (*P*. *laurifolia* × *P*. *deltoides*), sample 1 and 2; P.×RA—*P*. × *rasumowskyana* (*P. laurifolia × P. × wobstii*); P.SU—*P*. *suaveolens*; P.SI—*P*. *simonii*; P.TA.1, P.TA.2—*P*. *tacamahaca*, sample 1 and 2). The main flavonoids in this group included pinobanksin, chrysin, pinocembrin, pinocembrin chalcone, pinobanksin 3-acetate, and pinostrobin chalcone; the main hydroxycinnamic acid monoesters were caffeic acids derivatives (butyl or isobutyl isomer I, 2-methyl-2-butenyl, 3-methyl-2-butenyl, 3-methyl-3-butenyl ester, benzyl, phenethyl). Most of these substances, except caffeic acid butyl or isobutyl ester isomer I, were present in all EtOH in this group.

The next group gathered two extracts with the domination of hydroxycinnamic acid monoesters. Both of them originated from *P. nigra* (P.N.1 and P.N.2). The main hydroxycinnamic acid monoesters in this group were derivatives of caffeic acid (2-methyl-2-butenyl, 3-methyl-2-butenyl, 3-methyl-3-butenyl ester, benzyl, phenethyl) and metoxycinnamic acid cinnamyl esters. Both samples also contained average signals of flavonoids (mainly pinocembrin and pinostrobin chalcone). It is essential to point out that these samples did not contain pinobanksin 3-acetate (the main flavonoid of other investigated samples) and had only traces of non-esterified pinobanksin when another sample (also classified as *P. nigra*) contained these flavanols at high concentrations. In the literature, both flavonoids are frequently reported in *P*. *nigra* buds [17]. P.N.1 and P.N.2 were introduced to Arboretum from an old tree stand (Dęblin, on-Vistula river, Poland) and were previously evaluated as genetically pure *P. nigra.* As already mentioned in the Introduction, *P. nigra* easily crosses with available poplar species [1,4]. Therefore, we hypothesize pinobanksin and pinobanksin 3-acetate presence in the so-called *P. nigra* bud sample is a result of *P. nigra* hybridization, unnoticeable by morphological examination methods. Another possibility is that the P.N.3 sample originates from a specific phenotype (chemotype) of *P*. *nigra*.

The fourth group was hydroxycinnamic acid glycerides, mainly represented by *P*. *lasiocarpa* (P.LAS), *P*. *wilsonii* (P.WIL), and *P*. × *wilsocarpa* (*P*. *wilsonii* × *P*. *lasiocarpa*, P.×WCA). The main components in this group included 2-acetyl-1,3-di-caffeoylglycerol, 2-acetyl-1-caffeoyl-3-*p*-coumaroylglycerol, and 2-acetyl-1,3-di-*p*-coumaroylglycerol. In this group, P.LAS exhibited the strongest relative abundance of these compounds. Moreover, EtOH extracts in this group contained relatively low amounts of flavonoids, while hydroxycinnamic acid monoesters were absent. Hydroxycinnamic acid glycerides are specific markers of *P*. *lasiocarpa* from Asian great leaf poplar buds (section *Leucoides*) [34] as well as aspen poplars (Eurasian *P*. *tremula* [16,23] and American *P*. *tremuloides* [35]). Apart from P.LAS, P.WIL, and P.×WCA, the rest of the bud extracts contained only trace signals of these compounds (mainly monocaffeoylglycerol and rarely other glycerol esters).

The last group was a (five) mixed type, including five EtOH extracts (P.M×P.TRI.—*P*. *maximowiczii* × *P*. *trichocarpa*; P.RO—*P*. *trichocarpa* clone ‘Rochester’; P.TA×P.TRI.1, P.TA×P.TRI.2—*P*. *tacamahaca* × *P*. *trichocarpa*, sample 1 and 2; P.TRI—*P*. *trichocarpa*). It is worth pointing out that all samples in this group were *P*. *trichocarpa* and its crossbreed specimens with *P*. *maximowiczii* and *P*. *tacamahaca*. The lack of parent species—P.MAX, P.TA.1, and P.TA.2 in the (five) mixed group—led to a hypothesis that the impact of *P*. *trichocarpa* on secondary metabolites production in its hybrids is more substantial than the impact of other parent species. P.M×P.TRI, P.RO, P.TRI, and P.TA×P.TRI.2 revealed average and strong signals of *p*-coumaric acids, 2′,6′-dihydroxy-4′-methoxy dihydrochalcone and *p*-coumaric acid cinnamyl ester. Moreover, EtOH extracts also exhibited the presence of substances tentatively identified as balsacones (dihydrochalcones with additional phenylpropyl units). These components were previously isolated from *P*. *balsamifera* [36] but were absent in P.BA.EtOH extract. Readers need to remember that *P*. *trichocarpa* is sometimes classified as a subspecies of *P*. *balsamifera* [2]. The rest of the components in the mixed type were more varied. P.RO had strong signals of pinocembrin, pinocembrin dihydrochalcone, and pinobanksin 3-acetate, while the rest of the five samples had no more eye-catching components.

In summary, batch negative mode LC-MS analysis of poplar bud extracts revealed the dominance of polyphenols’ peaks with patterns specific enough to distinguish six groups. This method can be considered a promising strategy for poplar bud fingerprinting. Moreover, all EtOH extracts had stronger signals of polyphenols (from one to three relative levels of difference between considerable components; see Supplementary Table S1) than W/E, which can be practical information for further studies.

It is worth adding that buds of P.BA, P.KOM, P.MAX, P.M × P.TRI, P.N.3, P × PE.2, P.RO, P × RA, P.RO, P.TA.1, P.TA × P.TRI.1, P.TA × P.TRI.2, and P.TRI were relatively big (up to 4 cm), contained a lot of resins (organoleptic tests), and provided high extraction yield (>39% per dry mass of buds for EtOH extracts; see Supplementary Table S1. Finally, their extracts revealed strong signals of polyphenols. Therefore, they may be utilized as a source of specific components or extracts rich in polyphenols.

### 2.2. Antimicrobial Properties of Extracts

The antimicrobial properties of extracts are presented in Table 2 (Comparison of antimicrobial effect of ethanol and water/ethanol extracts of *Populus* buds). Performed research included determination of MIC (minimal inhibitory concentration) and MBC or MFC (minimal bactericidal/fungicidal concentration) as well as MBC/MIC or MFC/MIC ratio. Both extract types (EtOH and W/E) revealed relatively higher activity against Gram-positive bacteria and fungi than against most Gram-negative ones. Only *Helicobacter pylori* violated this rule; therefore, it was described separately below. Similar profiles of activity were already reported for poplar propolis [19,20,33] as well as buds of *P*. *nigra* [19,20], *P*. *balsamifera* [11,37], *P*. *tremula* [19,20], and *P*. *tremuloides* [35].

#### 2.2.1. Activity against Gram-Negative Strains

Most lyophilized poplar bud extracts (50/52) exhibited MIC (minimal inhibitory concentration) 1000 and >1000 µg/mL against *Escherichia coli*, *Salmonella* Typhimurium, *Proteus mirabilis,* and *Klebsiella pneumoniae*. MICs determination performed for EtOH extracts showed MIC values from 500 (only P.M × P.B vs. *P*. *mirabilis*) to >1000 µg/mL values. For this reason, the screening method was modified for W/E, and if MIC was >1000 µg/mL, MBC was not tested due to the expected unattractive high value and low activity. In the literature, ethyl acetate extracts exhibited MIC = 250 µg/mL (*P*. *nigra*) and 500 µg/mL (*P*. *tremula*) against *P*. *aeruginosa* and >5000 µg/mL (*P*. *nigra* and *P. tremula*) against *E*. *coli* [19]. In the case of methanol extracts of *P*. *nigra*, *P*. *alba*, and *P*. *tremula*, MIC > 4000 µg/mL were observed against *E*. *coli*, *K. pneumoniae*, *P. mirabilis*, *P*. *aeruginosa*, and *S. enterica* serovar Typhimurium [21]. In the case of *P. balsamifera*, weak activity against Gram-negative strains was exhibited in the disc-diffusion test [11].

Low activity against Gram-negative species may be a general rule for *Populus* bud extracts. It may result from components non-specifically eliminated via efflux pumps in many Gram-negative species [38], as well as differences in the structure of Gram-positive and Gram-negative cell barriers [39].

#### 2.2.2. Activity against Gram-Positive Strains

The tested Gram-positive bacteria strains included *Staphylococcus aureus*, *S. epidermidis*, *Micrococcus luteus*, *Bacillus subtilis*, *B*. *cereus*, and *Enterococcus faecalis*. Comparison of MIC with MBC in pairs of EtOH and W/E extracts exhibited that the MBC/MIC ratio was usually equal to or lower than four for most strains and samples. These results showed that EtOH and W/E exhibit rather bactericidal than bacteriostatic effects. The bactericidal effect may result from a multifactorial mechanism of action. Antibacterial agents of poplar buds’ resins, propolis, and flavonoids are known for disrupting cytoplasmic membrane function, inhibiting nucleic acid synthesis, and inhibiting the energy metabolism of bacterial strains [40]. Similar effects on bacterial cell membranes were also caused by caffeic acid monoesters, especially CAPE (caffeic acid phenethyl ester), which is the most investigated [41]. Potentially, ingredients that disrupt cell barrier function and stability may facilitate the penetration of active ingredients across the cell barrier. Active components may cause damage to bacterial cells in different ways, e.g., by increasing oxidative stress [42]. Finally, a bacterial cell cannot be repaired and undergoes lysis.

There were noted differences between the activity of EtOH and W/E extracts in most cases. Moreover, EtOH extracts usually showed a stronger antibacterial activity than W/E. Among all extracts, the weakest activity was exhibited by extracts belonging to the hydroxycinnamic acid glycerides group (P.LAS, P.WIL, and P. × WCA; MICs from 250 to ≥1000 µg/mL against all Gram-positive strains). The remaining extracts exhibited varied MICs (from 31.3 to ≥1000 µg/mL) against different strains. In the case of propolis, previous research showed that the presence of hydroxycinnamic acid glycerides may be connected with weaker antimicrobial activity for 70% aqueous ethanol extracts [20,43]. On the other hand, Isidorov et al. [19] showed that ethyl acetate extracts of *P*. *tremula* buds, rich in hydroxycinnamic acid glycerides, had better MIC against Gram-positive bacteria (*Staphylococcus schleiferi*, *S*. *aureus*, *B. cereus*, and *B*. *thuringiensis*). In another research, better activity of *P*. *tremula* methanolic extracts was exhibited against *S. aureus* and *B. cereus* [21]. It seems important to point out that both reports revealed only a twofold difference between the MIC of *P*. *tremula* and *P. nigra*. In the case of disc-diffusion studies of *P*. *tremula* and *P. nigra* buds’ antibacterial activity, it was shown that the more potent activity of *P*. *tremula* extracts was not a rule, and sometimes *P. nigra* was a better antibacterial agent [20]. In summary, the impact of hydroxycinnamic acids glycerides on whole extracts’ activity against Gram-positive bacteria is somewhat complex and probably depends on the presence and concentration of other components and their interactions.

**Figure 2:**
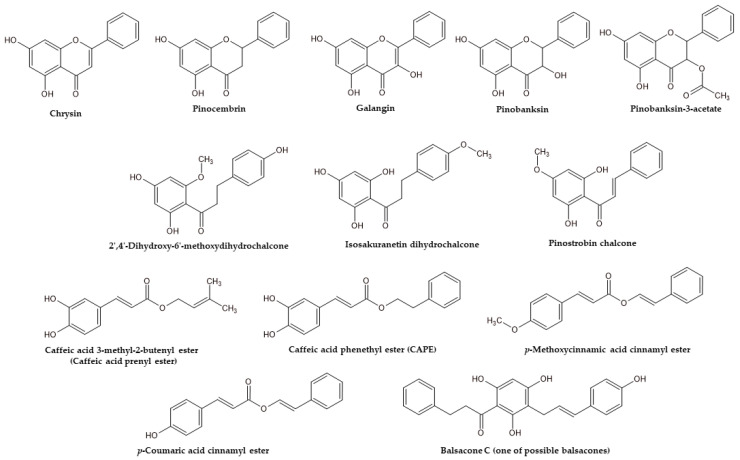
Antimicrobial agents of *Populus* buds.

Among all poplar bud extracts, the most potent antibacterial agent against Gram-positive bacteria was EtOH extract from P.TA×P.TRI.2 (MIC = 7.8 µg/mL vs. *S. epidermidis* and *M*. *luteus*, 15.6 µg/mL vs. *E*. *faecalis* and 31.3 µg/mL *S*. *aureus*, *B*. *cereus* and *B*. *subtillis*). Among others, only the EtOH extract of P.TA×P.TRI.2 exhibited stronger activity against *B*. *cereus* (MIC = 15.6 µg/mL). Other strong antibacterial agents included EtOH extracts of P.M × P.TRI, P.TA × P.TRI.1, P.TRI, and P.RO, as well as the W/E extract of P.TA × P.TRI.2, possessing significant activity against all tested Gram-positive strains (MIC ≤ 62.5 µg/mL). The potent extracts contained *p*-coumaric acid (P.TA × P.TRI.2, P.TRI, P.RO), pinocembrin (P.RO), isosakuranetin dihydrochalcone (P.TA × P.TRI.2), pinocembrin dihydrochalcone (P.TA × P.TRI.2), 2′,6′-dihydroxy-4′-methoxydihydrochalcone (P.TA × P.TRI.1, P.TA × P.TRI.2, P.TRI, P.RO), *p*-coumaric acid cinnamyl ester (P.TA × P.TRI.2, P.TRI, P.RO), and different components, tentatively identified as balsacones (P.TA × P.TRI.1, P.TA × P.TRI.2, P.TRI, P.RO). So far, it has been reported that balsacones exhibit activity against *S*. *aureus* strains [44]. Therefore, the presence of balsacones was the main difference between the mixed group and the rest of the phytochemical groups; these components may play an important role in the antibacterial effect. The structures of balsacones and other compounds hypothetically responsible for high anti-Gram-positive bacteria activity are presented in Figure 2. In summary, *Populus* buds classified in a mixed group exhibited the most potent activity against Gram-positive bacterial strains (see Table S1). Moreover, all of them were clones of *P*. *trichocarpa* and its crossbreed species.

Research on propolis showed that significant amounts of *p*-coumaric acid are connected with lower antimicrobial activity [20,45]. *p*-Coumaric acid alone showed antimicrobial activity but was an inferior antimicrobial agent to propolis flavonoids [46]. Moreover, the high amounts of *p*-coumaric acid were correlated with a low abundance of flavonoids. As a result, it was suggested that the weaker activity of propolis with a high concentration of *p*-coumaric acid was caused by the lower amounts of flavonoids [20]. In the literature, antibacterial effects against Gram-positive bacteria of propolis extracts were usually connected to the presence of some caffeic acid esters, such as CAPE [45], and flavonoids (pinobanksin 5-methyl ether, pinobanksin, chrysin, galangin, and pinobanksin 3-acetate) [43]. Most of these components were present in P.TA × P.TRI.2 and the other most potent antibacterial agents (P.M × P.TRI, P.TA × P.TRI.1, P.TRI, P.RO). However, their signals were weaker than in samples with lower activity (flavonoid, hydroxycinnamic monoesters, and flavonoid + hydroxycinnamic monoester types). For this reason, it may be suspected that these components do not play a decisive role in the antibacterial effect of *Populus* bud extracts. In our opinion, the final activity against Gram-positive strains results from different interactions between specific components.

#### 2.2.3. Activity against *Candida* spp.

Determination of antifungal activity included a screening of MIC and MFC against three *Candida* species (*C*. *albicans*, *C*. *glabrata*, and *C*. *parapsilosis*). Most extracts (EtOH and W/E) exhibited moderate activity (MIC ≥ 125 µg/mL and ≤500 µg/mL). Weak activity (MIC ≥ 1000 µg/mL) was observed for EtOH extracts of P.LAS, P.LAU, P.WIL, and P. × WCA, as well as for W/E extracts of P.WIL and P. × WCA. Good activity (MIC = 62.5 µg/mL) was only presented by EtOH extracts of P.CA, P. × PE.1, P.TRI (vs. *C*. *parapsilosis*), P.N.3 (vs. *C*. *glabrata*), and P.RO (vs. *C*. *glabrata* and *C*. *parapsilosis*) as well as W/E of P.N.3 (vs. *C*. *glabrata*) and P.TRI (vs. *C*. *glabrata* and *C*. *parapsilosis*). Observed MICs against *Candida* strains were generally higher than against Gram-positives. Moreover, most samples had a fungicidal rather than a fungistatic effect. In the literature, *P*. *nigra* bud extracts exhibited MICs = from 62.5 µg/mL (ethyl acetate extract) [19] to 1000 µg/mL (methanol extract) against *C*. *albicans*, while *P. tremula* buds were inactive (ethyl acetate extract) [19] or exhibited mild activity (methanol extract) (MIC = 500–1000 µg/mL) [19]. Research on propolis from these two species (*P*. *tremula* and *P*. *nigra*) demonstrated similar results [43]. A comparison of the experimental data with the literature allows us to suspect that apolar extracts of *Populus* buds should exhibit better activity against fungi polar extracts. However, data on the antifungal activity of propolis and *Populus* buds are not as widely available as for antibacterial activity. For these reasons and promising activity, further research is required (especially regarding the mechanism of action).

#### 2.2.4. Activity against *Helicobacter pylori*

Most of the EtOH extracts exhibited good activity (MIC ≤ 62.5 µg/mL) against *H. pylori*; only the activities of EtOH extracts from P.LAS, P.WIL, and P. × WCA were moderate (MIC from 250 to >1000 µg/mL). This suggests that a higher concentration of apolar flavonoids and phenolic acid monoesters increases the anti-*Helicobacter* activity of extracts. For propolis, it was proven that multiple polyphenolic substances can be connected with notable activity against *H. pylori* [46]. As listed previously, they are pinobanksin, pinobanksin 5-methyl ether, pinobanksin 3-acetate, chrysin, pinocembrin, and galangin, as well as *p*-methoxycinnamic acid cinnamyl ester [46]. Except for pinobanksin 5-methyl ether, these components were abundant in active extracts of *Populus* buds, and their presence correlated with anti-*Helicobacter* activity. Antibacterial agents of poplar buds may attack the cell barrier, disrupt metabolism, inhibit energy production, and cause oxidative stress in bacterial cells. The anti-*Helicobacter* effect of flavonoids was documented [47]. Krzyżek et al. [48] proved that myricetin slows the process of transformation into coccoid forms, reduces biofilm formation of *H. pylori*, and exhibits additive effects with clarithromycin and metronidazole. Other anti-*Helicobacter* properties were recorded by González et al. [49]. In their research, flavonoids such as chrysin inhibited the function of HsrA (one of the transcriptional regulators essential for cell viability) [49]. Moreover, it was proven that flavonoid-rich propolis extracts [46] and single flavonoids isolated from propolis [49] inhibit the urease of *H. pylori*. Urease increases the low pH of gastric juice, which allows the survival of *H. pylori*. This effect may be potentially used in anti-*Helicobacter* therapies. From the clinical point of view, it is also important that Korean propolis exhibits an anti-inflammatory effect on gastric mucous membranes (infected gastric mucosal injury mice model) [50]. In summary, all earlier propolis research suggests that poplar bud extracts may be used in anti-*Helicobacter* therapy in the future. Herewith, we report the observations in this field systematically.

## 3. Materials and Methods

### 3.1. Populus Buds and Chemicals

Poplar buds samples were collected in Spring 2015 from Szczodre, Poland (*P. nigra*, sample code: P.N.3) and Botanical Garden of the Adam Mickiewicz University in Poznań (*P*. *suaveolens* sample code: P.SU) as well as from Arboretum of Institute of Dendrology, Polish Academy of Sciences in Spring 2021 (*P*. *balsamifera*, *P*. *cathayana*, *P*. *deltoides*, *P*. *deltoides* × *P*. *nigra*, *P*. ‘Eridano’ (*P*. *deltoides* × *maximowiczii* clone Eridano), *P*. *komarowii*, *P*. *laurifolia*, *P*. *lasiocarpa*, *P*. *maximowiczii*, *P*. *maximowiczii* × *P*. *berolinensis*, *P*. *maximowiczii* × *P*. *trichocarpa*, *P*. *nigra*, sample 1; *P*. *nigra*, sample 2; *P*. *petrowskiana*, sample 1; *P*. *× petrowskiana (P. laurifolia × P. deltoides)*, sample 2; *P*. × *rasumoskowiana*, *P*. *trichocarpa* ‘Rochester’, *P*. *simonii*, *P*. *tacamahaca* sample 2, *P*. *tacamahaca* sample 2, *P*. *tacamahaca* × *P*. *trichocarpa* sample 1, *P*. *tacamahaca* × *P*. *trichocarpa* sample 2, *P*. *trichocarpa*, *P*. *wilsoni* and *P*. × *wilsocarpa*, samples codes: P.BA, P.CA, P.DE, P.DE × P.N, P.ERI, P.KOM, P.LAU, P.LAS, P.MAX, P.M × P.B, P.M × P.TRI, P.N.1, P.N.2, P. × PE.1, P. × PE.2, P. × RA., P.RO, P.SI, P.TA.1, P.TA.2, P.TA × P.TRI.1., P.TA.2 × TRI, P.TRI, P.WIL P. × WCA, respectively). Samples P.N.1, P.LAS, P.SU, P.WIL, and P. × WCA were collected from mature specimens while the rest were obtained from coppices. Samples from natural environment (Szczodre) were identified by author (P.O.) and originated from former *Populus* plantation in Szczodre (part of forest now). Plants originating from Botanical Garden of the Adam Mickiewicz University in Poznań and Arboretum of Institute of Dendrology, Polish Academy of Sciences originated from long-time collection and were marked according to the latest tree and shrub internal catalogues of these institutions. After collection, fresh plant material was dried at in room temperature in a dry, shady room with free airflow. The initial drying process took three weeks. Next, initially, dried buds were ground in a mill and again dried for a week at room temperature and free airflow in a dry, shady room. Due to the very sticky form of plant material, it was not sifted thought sieves. Full drying took four weeks. Before extraction, dried, ground plant material was stored in sealed containers under −20 °C.

LiChrosolv^®^ hypergrade eluents for UHPLC-MS/MS and UHPLC-DAD analysis (acetonitrile, water, and methanol) were purchased from Merck company (Darmstadt, Germany). Mueller–Hinton agar and Sabouraud agar were obtained from Oxoid (Hampshire, UK).

Standards of acacetin, apigenin, chrysin, kaempferol, kaempferide isorhamnetin, isosakuranetin, luteolin, genkwanin, pinocembrin, pinocembrin chalcone, pinocembrin dihydrochalcone, pinobanksin, pinostrobin, quercetin, rhamnetin, sakuranetin, tectochrysin were purchased from Extrasynthese (Genay, France) while caffeic acid, caffeic acid phenethyl ester (CAPE), ferulic acid were obtained from Sigma-Aldrich (Saint Louis, MO, USA).

### 3.2. Preparation of Populus Bud Extracts

The extraction process was based on our previous research on propolis and poplar buds [16,20]. Ground plant material was extracted with ethanol (96%, *V*/*V*) or with 50/50 ethanol in water (*V*/*V*) at the ratio of 1:10 (1.0 g of buds per 10.0 mL of solution). The extraction yield was provided in Supplementary Table S1. Extraction was performed in an ultrasonic bath (Sonorex, Bandelin, Berlin, Germany). Extraction conditions were set to 20 °C (initial temperature) for 15 min and 756 W (90% of ultrasonic bath power). The process was repeated thrice (total extraction time was 45 min). The temperature during all the process did not exceed 45 °C. Obtained extracts were stored at room temperature for 12 h for stabilization purposes (precipitation of potentially co-extracted wax). Next, extracts were filtered through the Whatman No. 10 paper (Cytiva, Marlborough, MA, USA), and ethanol was evaporated under reduced pressure. Next, extracts were frozen and lyophilized in Alpha 2-4 LD Plus lyophilizer (Christ, Osterode am Harz, Germany). Extraction yield was evaluated as a gram of lyophilized extract per gram of dried buds (see Supplementary Table S1).

### 3.3. UHPLC-DAD-MS/MS Profiling of Populus Bud Extracts

UHPLC analyses were performed as previously described [33] with a Thermo Scientific UltiMate 3000 system (Thermo Scientific™ Dionex™, Sunnyvale, CA, USA), coupled with an autosampler and DAD detector recording spectral data in the 200–600 nm range and monitoring at 280, 320, and 360 nm. UHPLC-MS/MS was carried out using a Compact ESI-qTOF MS/MS detector (Bruker Daltonics, Bremen, Germany). MS detector was used in electrospray negative mode. Conditions of analysis were ion source temperature was set to 210 °C, nebulizer gas pressure to 2.0 bar, and dry gas (nitrogen) flow to 8.01 L/min. The capillary voltage was 4.5 kV. The collision energy was set to 8.0 eV. Internal calibration was obtained run by run with a 10 mM sodium formate solution. For ESI-MS/MS experiments, collision energy was set at 35.0 eV, and nitrogen was used as collision gas. The scan range was set between 30 and 1300 *m*/*z*.

Identification of components was based on several parameters, such as retention times of chromatographic peaks and UV spectra, calculated formulas of deprotonated molecular ions, and MS/MS fragmentation spectra of deprotonated molecular ions. These values were compared with previous research (the same LC-ESI-UV-qTOF-MS/MS methods were used) [33], standards, and literature. The standards were used directly in current investigations (see list in Section 3.1) or in our previous research on propolis, a poplar resin mixed with beeswax [33]. For this reason, propolis may be partially used as a plant reference standard for poplar bud extracts. Literature about propolis LC-MS research is significantly more abundant than about poplar buds. For this reason, it is a valuable resource for comparisons.

Due to information collected from the literature, four levels of identification confidence were obtained: A (comparison of UV and MS/MS spectra with standards; the highest level of confidence), B (comparison of MS/MS and/or UV spectrum with literature; good level of confidence), C (component was identified according to deprotonated molecular ion formula and prediction from MS/MS spectra detected in *Populus* genus in literature, but there are no sufficient MS and UV data; average/weak level of confidence), and D (component was identified according to deprotonated molecular ion and prediction from MS spectra, but there are no sufficient MS/MS and UV data and substances were not reported in *Populus* genus literature; the weakest level of confidence). In the case of high-resolution mass spectrometry and calculations of the formulas, those with errors higher than 5 ppm were disqualified.

Semi-quantitative analysis was based on the relative abundance of components in the UV chromatogram (280 nm) and MS chromatogram. Relative abundance of most constituents was obtained due to deprotonated molecular ion intensity in a single chromatographic peak (IDMI). Due to received intensity, eight levels of relative abundance were created: tr (IDMI < 5 × 10^4^); + (5 × 10^4^ < IDMI > 1.5 × 10^5^); ++ (1.5 × 10^5^ < IDMI > 3 × 10^5^); +++ (3 × ^105^ < IDMI > 4.5 × 10^5^); ++++ (4.5 × 10^5^ < IDMI > 6.0 × 10^5^); +++++ (6.0 × 10^5^ < IDMI > 7.5 × 10^5^); ++++++ (7.5 × 10^5^ < IDMI > 1.0 × 10^6^); +++++++ (1.0 × 10^6^ < IDMI). Only pinostrobin and tectochrysin relative amounts were based on UV peak intensity due to their weak ionization in negative mode.

### 3.4. Determination of Antimicrobial Activity

The propolis extracts dissolved in dimethylsulfoxide (DMSO) were screened for antibacterial and antifungal activities by microdilution broth method according to both the European Committee on Antimicrobial Susceptibility Testing (EUCAST) (www.eucast.org accessed on 3 January 2023) using Mueller–Hinton broth or RPMI with MOPS for growth of fungi, as we described elsewhere [51]. Minimal inhibitory concentrations (MICs) of the tested extracts were evaluated for the wide panel of the reference microorganisms, including Gram-negative bacteria (*Salmonella* Typhimurium ATCC 14028, *Escherichia coli* ATCC 25922, *Proteus mirabilis* ATCC 12453, *Klebsiella pneumoniae* ATCC 13883, *Pseudomonas aeruginosa* ATCC 9027 and *Helicobacter pylori*), Gram-positive bacteria (*Staphylococcus aureus* ATCC 25923, *Staphylococcus epidermidis* ATCC 12228, *Micrococcus luteus* ATCC 10240, *Bacillus subtilis* ATCC 6633, *Bacillus cereus* ATCC 10876, and *Enterococcus*. *faecalis* ATCC 29212), and fungi (*Candida glabrata* ATCC 90030, *Candida albicans* ATCC 102231, *Candida parapsilosis* ATCC 22019). The sterile 96-well polystyrene microtitration plates (Nunc, Roskilde, Denmark) were prepared by dispensing 100 μL of appropriate dilution of the tested extracts in broth medium per well by serial twofold dilutions to obtain final concentrations of the tested extracts ranging from 1000 to 1.95 mg/L The inocula were prepared with fresh microbial cultures in sterile 0.85% NaCl to match the turbidity of 0.5 McFarland standard and were added to wells to obtain final density of 5 × 10^5^ CFU/mL for bacteria and 5 × 10^4^ CFU/mL for yeasts (CFU, colony forming units). After incubation (35 °C for 24 h), the MICs were assessed visually as the lowest concentration of the extracts that shows complete growth inhibition of the reference microbial strains. Appropriate DMSO control (at a final concentration of 10%), a strain growth control (inoculum without the tested extracts), and medium sterility control (the tested extracts without inoculum) were included on each microplate. The MIC for *H. pylori* ATCC 43504 was determined using a twofold microdilution method in MH broth with 7% of lysed horse blood at extract concentration ranging from 1000 to 1.95 mg/L with bacterial inocula of 3 McFarland standard. After incubation at 35 °C for 72 h under microaerophilic conditions (5% O_2_, 15% CO_2_, and 80% N_2_), the growth of *H*. *pylori* was visualized with the addition of 10 μL of 0.04% resazurin to each well. The MIC endpoint was recorded after 4 h incubation as the lowest concentration of extract that completely inhibits growth [52].

Minimal bactericidal concentration (MBC) or minimal fungicidal concentration (MFC) was obtained by a culture of 5 mL from each well that showed through growth inhibition, from the last positive one, and from the growth control onto recommended agar plates. The plates were incubated at 35 °C for 24 h for all microorganisms except *H*. *pylori*, which was incubated for 72 h in microaerophilic conditions. The MBC/MFC was defined as the lowest extract concentration without the growth of microorganisms. The MBC/MIC ratios were calculated to determine the bactericidal or bacteriostatic effect of the assayed extract. Vancomycin, ciprofloxacin, metronidazole, and nystatin were the reference drugs for Gram-positives, Gram-negatives, *H. pylori*, and yeasts, respectively. The experiments were repeated in triplicate. Representative data are presented.

## 4. Conclusions

Most of the 54 analyzed bud extracts from various poplar taxons were potent antibacterial agents against Gram-positive bacterial strains and *Helicobacter pylori*. Moderate activity was exhibited against *Candida* species while nonsignificant activity was demonstrated against most Gram-negative bacterial strains. The main identified or tentatively identified constituents of active extracts were flavonoid aglycones, hydroxycinnamic acid monoesters, and specific glycerides.

The good activity against Gram-positive bacterial strains and *H. pylori* makes poplar bud extracts an excellent candidate for the treatment of external infections caused by Gram-positive cocci and *Candida* spp., as well as for the treatment of stomach mucous membrane infection caused by *H*. *pylori*. Moreover, poplar buds may also serve as a source of specific components and extracts rich in bioactive polyphenols.

## Figures and Tables

**Table 1:** Identification of components in *Populus* bud extracts by LC-UV-ESI-qTOF-MS/MS.

No.	Component	RT (min)	UV_max_ (nm)	[M−H]^−^	Base MS/MS Peak	Secondary MS/MS Peaks *m/z* (A (%))	[M−H]^−^ (Formula)	Error (mDa)	Error (ppm)	RDB
1	Unidentified	0.84	ND/-	181.0721	-	-	C_6_H_13_O_6_	−0.3	−1.8	0.0
2	Unidentified	0.87	ND/-	195.0515	-	-	C_6_H_11_O_7_	−0.4	−2.3	1.0
3	Unidentified	0.89	ND/-	341.1090	113.1273	-	C_12_H_21_O_11_	−0.1	−0.2	2.0
4	Unidentified	1.22	290	191.0201	111.1597	-	C_6_H_7_O_7_	−0.4	−2.0	3.0
5	^C^ Leonuriside A	4.31	268	331.1029	123.1365	-	C_14_H_19_O_9_	0.5	1.5	11.0
6	^A^ Chlorogenic acid	6.55	*324	353.0876	191.1526	135.1 (62.42), 179.2 (50.00)	C_16_H_17_O_9_	0.2	0.5	8.0
7	^B^ Caffeoylglucose isomer I	8.24	328	341.0884	161.1265	133.1 (20.50), 135.1 (6.55), 179.1 (4.77)	C_15_H_17_O_9_	−0.6	−1.6	7.0
8	^B^ Caffeoylglucose isomer II	9.19	326	341.0889	161.0962	179.1 (56.51), 135.1 (55.31), 177.2 (32.50), 221.2 (31.58)	C_15_H_17_O_9_	−1.1	−3.1	7.0
9	^A^ Vanilline	9.36	273, 304sh	151.0397	108.1554	-	C_8_H_7_O_3_	0.3	2.1	5.0
10	^C^ Salicyl alcohol dihexoside	10.02	265	447.1508	269.2836	-	C_19_H_27_O_12_	0.0	0.0	6.0
11	^B^ Caffeoylglucose isomer III	10.24	321	341.0880	135.1517	179.2 (91.78), 161.1 (62.10), 221.2 (28.24), 177.1 (23.94)	C_15_H_17_O_9_	−0.2	−0.5	7.0
12	^C^*p*-Coumaric acid hexoside isomer I	10.54	314	325.0936	145.1268	117.1 (22.35)	C_15_H_17_O_8_	−0.7	−2.3	7.0
13	^B^ Catechin or Epicatechin	10.87	279	289.0724	123.1394	109.1 (87.26), 221.3 (35.68), 137.3 (36.96), 203.2 (30.66)	C_15_H_13_O_6_	−0.6	−2.2	9.0
14	^A^ Caffeic acid	11.46	323	179.0346	135.0449	107.0 (8)	C_9_H_7_O_4_	0.4	2.0	6.0
15	^B^ di-Caffeoylglycerol	11.91	323	415.1240	161.1272	415.4 (15.58), 179.1 (6.67), 133.2 (6.42)	C_18_H_23_O_11_	0.6	1.5	7.0
16	^D^ Feruloyl or isoferuloyl hexoside isomer I	12.14	328	355.1041	175.1718	160.1 (90.68)	C_16_H_19_O_9_	−0.7	−1.8	7.0
17	^B^ Methoxybenzaldehyde	12.64	*279	135.0451	92.3923	-	C_8_H_7_O_2_	0.0	0.0	5.0
18	^B^ Caffeoylglycerol	13.06	*320	253.0711	161.0743	133.2 (92.59), 135.1 (40.05)	C_12_H_13_O_6_	0.6	2.5	6.0
19	^C^ *p*-Coumaric acid hexoside isomer II	13.11	*314	325.0931	145.1625	119.2 (72.18), 163.1 (50.79), 205.1 (33.66)	C_15_H_17_O_8_	−0.2	−0.7	7.0
20	^D^ Feruloyl or isoferuloyl hexoside isomer II	13.36	*325	355.1037	134.1126	160.2 (95.34), 193.2 (87.81), 191.1 (66.31), 235.2 (62.90)	C_16_H_19_O_9_	−0.3	−0.8	7.0
21	^A^ *p-*Coumaric acid	14.42	309	163.0401	119.1668	93.1 (10.59)	C_9_H_7_O_3_	0.0	−0.1	6.0
22	^C^ 3,4,5-Trimethoxy-cinnamic acid	14.42	ND/-	237.0777	117.1037	145.1 (84.14)	C_12_H_13_O_5_	−0.8	−3.4	6.0
23	^B^ Salicortin	14.93	273	423.1303	123.1625	155.2 (61.54), 121.2 (49.91), 111.3 (45.59)	C_20_H_23_O_10_	−0.6	−1.4	9.0
24	^C^ 7-*O*-caffeoylsalirepin	15.18	324	463.1247	179.1438	161.2 (42.81), 135.2 (24.90)	C_22_H_23_O_11_	−0.1	−0.2	11.0
25	^A^ Ferulic acid	15.20	321	193.0509	134.1322	-	C_10_H_9_O_4_	−0.3	−1.5	6.0
26	^B^ Caffeic acid dihydroxypentyl or isopentyl ester isomer I	15.22	ND/-	281.1031	161.1330	135.1 (48.99), 133.3 (56.95), 179.2 (8.42)	C_14_H_17_O_6_	−0.1	−0.2	6.0
27	^A^ Isoferulic acid	15.71	322	193.0504	134.1696	-	C_10_H_9_O_4_	0.3	1.4	6.0
28	^C^ Taxifolin (Dihydroquercetin) isomer I	16.00	289	303.0517	125.0824	153.2 (21.31)	C_15_H_11_O_7_	−0.7	−2.3	10.0
29	^D^ Caffeic acid derivate	16.01	326	439.1613	161.1313	439.4 (17.06), 179.1 (11.23), 133.2 (5.41), 135.1 (3.72)	C_21_H_27_O_10_	−0.3	−0.7	8.0
30	^D^ Caffeic acid derivate	16.23	326	439.1612	161.1249	439.4 (19.80), 179.15 (10.97), 135.2 (5.13), 133.2 (4.22)	C_21_H_27_O_10_	−0.3	−0.6	8.0
31	^D^ Caffeic acid derivate	16.46	326	439.1612	161.1236	439.5 (28.26), 179.2 (11.69), 133.2 (3.78)	C_21_H_27_O_10_	−0.2	−0.6	8.0
32	^D^ Caffeic acid derivate	16.65	326	439.1601	161.1282	439.5 (41.10), 179.1 (6.95), 133.2 (3.52)	C_21_H_27_O_10_	0.9	0.2	8.0
33	Unidentified	16.67	ND	295.0828	161.1384	133.3 (55.74), 135.1 (37.87), 159.3 (11.28), 137.1 (8.71), 179.2 (6.48)	C_14_H_15_O_7_	−0.4	−1.5	7.0
34	^B^ Populoside isomer I	16.68	326	447.1297	161.1201	323.3 (29.72), 179.1 (16.49), 123.1 (9.47), 135.1 (6.61), 203.2 (3.00)	C_22_H_23_O_10_	−0.1	−0.2	11.0
35	^C^ Azelaic acid (Nonanedioic acid)	17.07	ND/-	187.0976	-	-	C_9_H_15_O_4_	−0.1	−0.3	2.0
36	^D^ *p*-Coumaric derivate	17.13	311	425.1459	145.1490	163.1 (45.05), 307.3 (31.45), 117.1 (21.30), 265.2 (19.12), 205.2 (18.31), 119.1 (15.12), 161.1 (6.99), 235.2 (5.94)	C_20_H_25_O_10_	−0.6	−1.3	8.0
37	^B^ Populoside isomer II	17.33	*326	447.1295	161.1565	179.1 (11.93), 123.1 (7.17), 121.1 (6.73), 135.1 (6.01), 133.3 (7.23), 323.3 (6.06), 447.3 (2.49)	C_22_H_23_O_10_	0.1	0.3	11.0
38	^C^ Eriodictyol (Dihydroluteolin) isomer	17.37	288	287.0560	139.2901	137.2 (24.06)	C_15_H_11_O_6_	0.1	0.5	10.0
39	Unidentified	17.40	*293	451.1249	121.1191	283.2 (46.50), 163.1 (12.29), 175.1 (4.64), 193.1 (3.37), 135.1 (3.40), 145.1 (2.41), 181.1 (2.31)	C_21_H_23_O_11_	−0.3	−0.7	10.0
40	^C^ Pinobanksin- or Naringenin 7-*O*-hexoside	17.57	286	433.1145	271.2293	165.1 (73.88), 433.4 (42.77), 253.2 (16.18), 243.2 (14.45), 225.2 (10.81), 313.3 (6.69), 227.2 (3.49), 197.2 (2.75), 151.2 (3.19), 241.2 (2.87)	C_21_H_21_O_10_	−0.5	−1.2	11.0
41	Unidentified	17.69	*283	451.1247	138.2292	121.1 (5.43), 413.1 (4.41), 163.1 (4.39), 151.1 (4.07), 181.2 (3.36), 405.3 (2.60), 193.2 (2.04)	C_21_H_23_O_11_	−0.1	−0.3	10.0
42	^C^ Vanilloyl-methyl-ketone	17.79	*286	193.0504	133.1891	-	C_10_H_9_O_4_	0.2	1.0	6.0
43	^C^ Taxifolin (Dihydroquercetin) isomer II	17.84	289	303.0519	151.1068	303.1 (17.51)	C_15_H_11_O_7_	−0.9	−2.8	10.0
44	^B^ Salireposide	18.27	ND/-	405.1192	242.2316	151.1 (78.89), 107.1 (27.34)	C_20_H_21_O_9_	−0.1	−0.3	10.0
45	Unidentified	18.42	ND/-	193.0872	-	-	C_11_H_13_O_3_	−0.2	−1.0	5.0
46	Unidentified	18.46	ND/-	465.1397	123.1096	155.2 (46.57)	C_22_H_25_O_11_	0.5	1.2	10.0
47	Unidentified	18.48	ND/-	511.1463	155.1086	123.1 (98.45), 111.1 (94.09), 137.1 (46.54), 109.1 (22.50), 405.4 (21.74), 121.1 (14.37)	C_23_H_27_O_13_	−0.5	−1.1	10.0
48	^B^ Eriodictyol (Dihydroluteolin)	18.83	291	287.0564	125.1152	177.2 (56.00), 152.4 (32.51), 107.4 (12.25), 259.3 (11.25), 213.2 (9.01)	C_15_H_11_O_6_	−0.2	−0.8	10.0
49	^B^ Isograndidentatin A	18.88	314	423.1651	145.1113	163.1 (12.51), 119.1 (6.64), 117.2 (5.33), 423.4 (6.05)	C_21_H_27_O_9_	1.0	2.3	8.0
50	^B^ Grandidentatin	19.30	312	431.1349	145.1447	163.1 (13.99), 123.1 (12.54), 307.4 (10.10), 119.1 (5.65), 121.1 (4.50), 187.1 (3.86)	C_22_H_23_O_9_	−0.1	−0.3	11.0
51	^B^ Caffeic acid dimethyl ether	19.48	324	207.0664	-	-	C_11_H_11_O_4_	−0.1	−0.5	6.0
52	^D^ Caffeic acid derivate	19.72	321	481.1716	161.1269	179.2 (38.23), 481.4 (14.79), 135.1 (6.74), 421.5 (6.68), 439.6 (5.39)	C_23_H_29_O_11_	−0.1	−0.1	9.0
53	^C^ Taxifolin 3′-methyl ether (Dihydroisorhamnetin)	20.29	ND/-	317.0673	152.1004	125.1 (38.44), 179.2 (16.01), 192.2 (11.21)	C_16_H_13_O_7_	−0.6	−2.0	10.0
54	^B^ Populoside isomer III	20.91	325	447.1301	179.1367	135.1 (26.66), 161.1 (24.41)	C_22_H_23_O_10_	−0.4	−0.9	11.0
55	^B^ Apigenin 7-*O*-glucoside (Apigetrin)	21.10	264, 309sh	431.0983	268.2682	431.3 (23.37), 240.1 (9.85), 211.2 (9.64)	C_21_H_19_O_10_	0.0	0.1	12.0
56	^B^ Diosmetin 7-*O*-rutinoside (Diosmin)	21.21	ND/-	607.1675	111.1002	155.2 (88.44), 123.1 (57.43), 161.1 (28.94), 137.1 (28.43), 109.1 (20.03), 423.5 (18.42), 405.4 (15.17), 299.3 (12.25), 113.1 (7.23), 561.5 (8.28), 101.3 (6.73), 143.1 (4.42), 93.2 (3.19), 317.5 (4.12), 165.2 (2.96), 449.3 (2.56), 159.2 (2.39)	C_28_H_31_O_15_	−0.6	−1.0	13.0
57	Unidentified	22.15	ND/-	385.1508	223.2634	208.2 (10.07), 152.1 (6.81), 205.2 (2.06)	C_18_H_25_O_9_	−0.4	−1.1	6.0
58	^D^ Caffeic acid derivate	22.22	328	489.1407	161.1159	179.2 (15.01), 123.1 (12.65), 133.2 (8.52), 135.1 (6.63), 489.3 (4.33)	C_24_H_25_O_11_	−0.5	−0.9	12.0
59	^B^ Caffeic ethyl ester	22.65	322	207.0664	133.3012	135.1162 (48.30), 161.0890 (16.11)	C_11_H_11_O_4_	−0.1	−0.5	6.0
60	^C^ Aromadendrin (Dihydrokaempferol)	23.08	288	287.0566	135.1329	151.1078 (15.43)	C_15_H_11_O_6_	−0.5	−1.8	10.0
61	^B^ Pinobanksin 5-methyl ether	23.40	287	285.0777	252.0429	224.0 (55.83), 138.0 (38.07), 241.0 (31.50), 165.0 (14.95), 239.1 (12.13), 195.0 (12.02), 151.0 (11.81), 213.1 (11.34), 267.1 (11.02), 285.1 (9.31), 136.0 (8.53), 107.0 (6.81)	C_16_H_13_O_5_	−0.8	−2.9	10.0
62	^C^ Kaempferol 3-methyl ether (Isokaempferide)	23.66	288	299.0553	227.1837	255.2 (69.84), 284.2 (9.97), 299.1 (7.83)	C_16_H_11_O_6_	0.8	2.6	12.0
63	Unidentified	23.83	ND/-	589.1563	122.2014	139.1 (89.97), 155.1 (75.54), 111.1 (52.83), 387.4 (44.27), 137.2 (32.78), 109.1 (26.60), 233.3 (22.16), 215.3 (20.13), 135.1 (16.17), 205.2 (6.94), 543.5 (8.81), 165.1 (4.14), 163.2 (3.15), 405.3 (2.79), 265.3 (2.13)	C_28_H_29_O_14_	0.0	−0.1	14.0
64	^B^ di-Caffeoylglycerol	24.58	320	415.1033	253.2248	161.1 (84.50), 179.1 (65.63), 135.1 (55.89)	C_21_H_19_O_9_	0.1	0.3	12.0
65	^A^ Quercetin	25.10	364, 270sh, 265	301.0353	151.0034	121.0 (29.41), 107.0 (22.18), 149.0 (14.01), 178.9 (13.92), 301.0 (7.58), 245.0 (6.32), 273.0 (5.48), 163.0 (4.87), 211.0 (3.84)	C_15_H_9_O_7_	0.1	0.3	11.0
66	^D^ Caffeic acid derivate	25.33	ND/-	445.1510	161.1296	445.5 (18.12), 179.2 (7.44), 135.2 (4.83)	C_23_H_25_O_9_	−0.6	−1.3	11.0
67	^A^ Luteolin	25.40	370	285.0412	133.1356	285.2 (83.77), 151.0 (33.21), 199.1 (15.09), 107.1 (12.83)	C_15_H_9_O_6_	−0.8	−2.7	11.0
68	^B^ Quercetin 3-methyl ether	26.85	255, 355	315.0497	271.0253	300.0 (71.14), 255.0 (42.89), 243.0 (22.59), 227.0 (2.55)	C_16_H_11_O_7_	0.2	0.5	11.0
69	^A^ Pinobanksin (Dihydrogalangin)	27.25	291	271.0615	197.0617	253.0 (89.28), 161.1 (67.51), 271.1 (56.26), 125.02 (53.39), 151.0 (30.14), 225.1 (24.71), 107.0 (23.97), 209.1 (16.07), 185.1 (15.86), 115.1 (15.08), 157.1 (14.43), 181.1 (14.14), 215.1 (11.83)	C_15_H_11_O_5_	−0.3	−1.1	10.0
70	^C^ Boropinic acid (Caffeic acid 3-methyl, 4-(3-methyl−2-buten−1-yl) ether)	28.52	ND/-	261.1133	145.1340	117.2 (48.10), 119.1 (14.84), 115.1 (3.56), 113.1 (3.07)	C_15_H_17_O_4_	−0.1	−0.2	7.0
71	^A^ Naringenin (Dihydroapigenin)	28.60	290	271.0612	119.1344	151.0 (43.37), 107.1 (21.94), 187.2 (10.00)	C_15_H_11_O_5_	0.0	0.1	10.0
72	^B^ Chrysin 5-methyl ether	28.80	ND/-	267.0662	224.1747	180.2 (92.97), 252.2 (26.27), 195.2 (15.00)	C_16_H_11_O_4_	0.1	0.3	11.0
73	^B^ Eriodictyol 3’-methyl ether (Homoeriodictyol) or Eriodictyol 4’-methyl ether (Hesperetin)	28.83	292	301.0723	152.0994	176.1 (55.71), 283.2 (57.41), 125.1 (50.48), 301.2 (51.90), 227.4 (33.57), 268.2 (25.56), 107.2 (17.38)	C_16_H_13_O_6_	−0.6	−1.9	10.0
74	^B^ 1-Caffeoyl−3-*p*-coumaroylglycerol	28.96	312	399.1085	163.1721	161.1 (48.44), 119.1 (48.96), 253.2 (46.08), 179.2 (25.62), 145.2 (24.73), 235.1 (20.40), 161.2 (10.73), 237.2 (8.31), 399.2 (5.30)	C_21_H_19_O_8_	0.0	0.1	12.0
75	^C^ Flavonoid	29.55	286	269.0822	150.0692	184.2 (88.87), 165.1 (80.74), 122.1 (55.22), 254.2 (50.90), 227.2 (38.24), 269.3 (20.13)	C_16_H_13_O_4_	−0.3	−1.0	10.0
76	^D^ Caffeic acid derivate	29.63	326	277.1084	135.1237	179.1 (11.92)	C_15_H_17_O_5_	−0.3	−0.9	7.0
77	^B^ Caffeic acid propyl or isopropyl ester	29.88	323	221.0824	133.7267	161.1 (22.49)	C_12_H_13_O_4_	−0.5	−2.2	6.0
78	^C^ Aromadendrin 7-methyl ether	30.36	287	301.0722	164.1585	151.1 (72.31), 136.1 (49.48), 134.3 (49.49), 108.1 (29.20), 242.2 (17.13), 286.2 (15.52), 214.6 (17.83)	C_16_H_13_O_6_	−0.5	−1.5	10.0
79	^C^ Naringenin chalcone	30.45	290	271.0617	125.1004	145.1 (23.55), 117.4 (8.59), 151.1 (6.04), 107.1 (3.82)	C_15_H_11_O_5_	−0.5	−1.7	10.0
80	^A^ Apigenin	30.51	267, 336	269.0457	117.0349	269.0 (52.06), 151.0 (39.01), 149.0 (25.91), 227.0 (12.66), 107.0 (11.48), 225.0 (10.59), 201.1 (7.44), 183.0 (6.40), 181.1 (5.14), 121.0 (4.92), 197.1 (2.28)	C_15_H_9_O_5_	−0.2	−0.7	11.0
81	^A^ Kaempferol	31.19	264, 365	285.0405	285.0400	239.0 (8.81), 187.0 (8.20), 185.0 (8.14), 229.0 (7.99), 159.0 (6.63)	C_15_H_9_O_6_	−0.1	−0.3	11.0
82	^B^ Caffeic acid hydroxyphenylethyl ester	31.47	*324	299.0922	135.1402	179.2 (24.97), 161.1 (5.41)	C_17_H_15_O_5_	0.3	1.0	10.0
83	^A^ Quercetin 3’-methyl ether (Isorhamnetin)	31.77	*253, 357	315.0509	300.1989	151.1 (26.66), 271.4 (11.37), 164.1 (7.61), 283.1 (6.12), 148.1 (5.64), 315.2 (5.60), 255.2 (4.65), 216.2 (3.38), 108.2 (2.95), 244.2 (2.60), 136.2 (2.55)	C_16_H_11_O_7_	0.1	0.3	11.0
84	^B^ Quercetin methyl ether isomer I	32.28	254, 367	315.0511	300.1857	151.1 (26.12), 271.3 (11.15), 164.1 (7.58), 283.1 (5.81), 216.3 (4.63)	C_16_H_11_O_7_	0.0	−0.1	11.0
85	^B^ Luteolin 5-methyl ether	33.03	265, 349	299.0549	255.0300	227.03 (59.96), 284.0 (15.07), 211.0 (6.11)	C_16_H_11_O_6_	−0.2	−0.7	11.0
86	^C^ Syringenin (sinapyl alcohol)	33.24	*296	209.0826	165.1925	125.1 (95.68), 123.2 (53.31), 124.3 (23.62)	C_11_H_13_O_4_	−0.7	−3.2	5.0
87	^B^ Caffeic acid butenyl or isobutenyl ester	33.73	ND/-	233.0818	133.3938	-	C_13_H_13_O_4_	0.1	0.5	7.0
88	^B^ Quercetin dimethyl ether isomer I	33.74	256, 354	329.0669	271.1688	299.2 (99.34), 243.2 (90.63), 285.4 (51.12), 257.2 (31.51), 314.2 (29.44), 227.2 (5.23), 215.2 (3.74), 199.2 (3.06), 255.1 (2.88)	C_17_H_13_O_7_	−0.2	−0.6	11.0
89	^D^ *p*-Coumaric acid derivate	33.81	ND	311.0923	119.1298	163.2 (30.72), 135.1 (7.31), 145.1 (3.83)	C_18_H_15_O_5_	0.2	0.7	11.0
90	^B^ 1,3-di-*p*-Coumaroylglycerol	33.98	312	383.1143	163.1491	119.1 (69.49), 145.1 (61.09), 117.2 (8.68), 219.2 (7.20), 237.2 (6.59), 383.4 (2.42)	C_21_H_19_O_7_	−0.7	−1.8	12.0
91	^B^ Galangin 5-methyl ether	34.41	*260, 350	283.0612	211.1796	239.2 (58.94), 283.3 (5.07), 268.2 (4.79)	C_16_H_11_O_5_	0.0	−0.1	11.0
92	^B^ 1,2-di-p-Coumaroylglycerol II	34.46	315	383.1137	163.1447	119.1 (78.80), 145.1 (70.92)	C_21_H_19_O_7_	−0.1	−0.2	12.0
93	^B^ Pinobanksin 5-methyl ether 3-acetate	34.69	288	327.0878	224.1781	267.2 (67.46), 252.2 (62.85), 285.2 (45.11), 239.5 (36.67)	C_18_H_15_O_6_	−0.4	−1.1	11.0
94	^B^*m*-Coumaric acid (3-Hydroxycinnamic acid)	35.01	311	163.0400	119.1298	163.2 (30.72), 135.1 (7.31), 145.1 (3.83)	C_9_H_7_O_3_	0.1	0.4	6.0
95	^B^ Pinobanksin 3-hydroxybutanoate isomer I	35.14	*292	357.0977	253.2321	271.2 (7.29), 197.2 (4.96), 209.3 (3.60)	C_19_H_17_O_7_	0.3	0.8	11.0
96	^B^ 2-Acetyl−1,3-di-caffeoylglycerol	35.23	326	457.1143	179.1554	161.1 (75.83), 235.2 (53.60), 135.1 (48.32), 295.3 (40.83), 457.3 (5.85), 397.3 (5.27)	C_23_H_21_O_10_	−0.3	−0.7	13.0
97	^B^ 1-Acetyl−2,3-di-caffeoylglycerol	35.73	325	457.1135						
98	^D^ Caffeic acid derivate	35.81	*326	291.1248	135.1307	179.1 (21.22), 269.1 (4.90)	C_16_H_19_O_5_	−1.0	−3.3	7.0
99	^B^ Quercetin methyl ether isomer II	36.15	ND/-	315.0883	164.0962	136.1 (51.83), 285.2 (39.16), 315.2 (22.41), 300.3 (14.01), 271.3 (12.30), 273.2 (10.71), 258.2 (7.41)	C_17_H_15_O_6_	−0.8	−2.7	10.0
100	^A^ Quercetin 7-methyl ether (Rhamnetin)	36.53	256, 353	315.0509	165.1079	121.1 (39.04), 300.2 (27.72), 151.1 (9.49), 272.2119 (6.69), 244.2 (4.72), 256.3 (3.45)	C_16_H_11_O_7_	0.1	0.4	11.0
101	^B^ Kaempferol methyl ether isomer I	36.68	ND/-	299.0563	284.1907	299.2 (7.35), 256.1 (5.21), 133.2 (5.23), 151.1 (2.37), 227.3 (2.53)	C_16_H_11_O_6_	−0.2	−0.7	11.0
102	^B^ Caffeic acid butyl or isobutyl ester isomer I	37.33	325	235.0978	133.5359	161.1 (41.79)	C_13_H_15_O_4_	−0.2	−1.0	6.0
103	^B^ Pinobanksin 3-hydroxybutanoate isomer II	37.55	293	357.0983	253.223	197.2 (4.80), 271.2 (4.93), 209.4 (2.89), 225.2 (2.52)	C_19_H_17_O_7_	−0.3	−0.8	11.0
104	Unidentified	37.74	288, 308sh	205.0877	117.388	145.2 (23.35)	C_12_H_13_O_3_	−0.7	−3.3	6.0
105	^B^ Caffeic acid butyl or isobutyl ester isomer II	37.96	325	235.0976	161.1424	135.1 (93.59)	C_13_H_15_O_4_	−0.1	−0.2	6.0
106	^C^ 2’,4’,6’-Trihydroxypentanophenone	38.89	286	209.0827	152.0951	124.1 (84.90), 194.2 (11.41), 148.1 (9.02), 111.1 (8.47), 96.2 (6.71), 179.1 (4.46)	C_11_H_13_O_4_	−0.7	3.4	5.0
107	^B^ Quercetin dimethyl ether isomer II	39.00	261, 357	329.0669	299.1970	271.2 (30.28), 314.2 (21.06), 285.2 (2.46)	C_17_H_13_O_7_	−0.3	−0.8	11.0
108	^B^ Caffeic acid 2-methyl−2-butenyl ester	39.19	325	247.0979	135.1258	161.1 (36.02), 179.1 (11.25)	C_14_H_15_O_4_	−0.4	−1.5	7.0
109	^B^ Quercetin dimethyl ether isomer III	39.41	ND	329.0670	299.1828	271.2 (39.73), 314.2 (27.41), 285.2 (12.61), 329.3 (2.26)	C_17_H_13_O_7_	−0.3	−0.9	11.0
110	^B^ Caffeic acid 3-methyl−2-butenyl ester (basic prenyl ester)	40.68	324	247.0979	134.2235	106.1 (6.32)	C_14_H_15_O_4_	−0.4	−1.7	7.0
111	^B^ Caffeic acid 3-methyl−3-butenyl ester (prenyl ester isomer I)	41.16	325	247.0977	134.2234	106.2 (5.64)	C_14_H_15_O_4_	−0.1	−0.4	7.0
112	^B^ Sakuranetin dihydrochalcone	41.56	285	287.0921	166.1295	181.2 (73.27), 152.1 (44.21), 124.1 (30.43), 226.2 (11.95), 193.1 (10.26), 254.2 (9.25), 178.2 (8.01), 139.1 (7.01), 93.1 (6.92), 189.2 (6.18), 150.2 (3.68), 269.3 (3.49)	C_16_H_15_O_5_	0.4	1.5	9.0
113	Unidentified	41.66	286	251.1648	-	-	C_15_H_23_O_3_	0.5	1.8	4.0
114	^B^ 2-Acetyl−1-caffeoyl−3-*p*-coumaroylglycerol	41.79	315	441.1197	163.1479	179.1 (85.75), 161.1 (42.10), 135.1 (40.85), 145.2 (39.56), 119.1 (35.73), 235.2 (27.59), 295.3 (14.64), 219.2 (7.31), 173.2 (6.88), 381.4 (7.79), 217.2 (4.50), 441.3 (4.75), 189.2 (3.80), 277.3 (2.86)	C_23_H_21_O_9_	−0.6	−1.3	13.0
115	^A^ Chrysin	42.12	267, 312sh	253.0505	253.0507	143.0 (41.53), 145.0 (21.10), 209.1 (14.10), 107.0 (13.33), 181.1 (8.16), 185.1 (6.19)	C_15_H_9_O_4_	−0.7	−2.8	11.0
116	^B^ Caffeic acid benzyl ester	42.55	324	269.0818	134.1302	161.0 (22.96), 137.0 (4.03)	C_16_H_13_O_4_	−0.3	−1.1	10.0
117	^B^ 2-Acetyl−3-caffeoyl−2-feruloylglycerol	42.59	314	471.1290	193.1743	179.1 (91.94), 135.1 (38.51), 161.1 (37.37), 175.1 (35.75), 235.2 (23.96), 295.2 (15.68), 149.1 (9.17), 411.3 (9.38), 173.2 (7.01), 249.2 (6.57), 471.5 (7.85), 217.1 (5.91), 367.2 (4.13), 189.2 (3.32), 117.2 (3.04), 277.3 (2.88)	C_24_H_23_O_10_	0.7	1.5	13.0
118	^D^ Flavonoid	42.60	ND/-	285.0772	119.1332	165.28 (29.97), 150.4 (14.74), 121.1 (5.58), 122.1 (5.43), 269.3 (5.59), 97.1 (3.07), 136.2 (2.95), 177.2 (2.52)	C_16_H_13_O_5_	−0.3	−1.1	10.0
119	Unidentified	42.71	313	217.0869	117.1863	145.2 (2.80)	C_13_H_13_O_3_	0.1	0.4	7.0
120	^A^ Pinocembrin	43.07	289	255.0666	171.0464	151.0 (80.69), 255.1 (75.17), 213.1 (74.89), 145.1 (70.09), 107.0 (52.59), 185.1 (34.69), 169.1 (24.91), 211.1 (23.68), 164.0 (17.93), 187.1 (16.78), 136.0 (16.34)	C_15_H_11_O_4_	−0.2	−0.8	10.0
121	^A^ Pinocembrin chalcone	43.30	342	255.0668	171.2600	151.1 (61.32), 107.3 (40.48), 145.1 (29.50), 255.2 (25.04), 169.2 (23.80), 213.1 (21.71), 211.2 (14.01), 164.1 (9.13), 136.3 (7.29), 187.2 (6.32), 143.2 (4.35), 193.3 (3.34)	C_15_H_11_O_4_	−0.5	−2.0	10.0
122	^A^ Naringenin 7-methyl ether (Sakuranetin)	43.32	289	285.0768	119.1265	165.1 (18.08)	C_16_H_13_O_5_	0.3	0.7	10.0
123	Unidentified	43.88	ND	223.0985	179.2917	139.1 (70.78), 137.1 (40.96), 115.2 (8.88)	C_12_H_15_O_4_	−0.9	−3.9	5.0
124	^A^ Naringenin 4’-methyl ether (Isosakuranetin)	44.31	290	285.0773	124.1060	139.1 (64.17), 145.1 (42.28), 148.1 (8.73), 165.1 (4.71)	C_16_H_13_O_5_	−0.4	−1.6	10.0
125	^A^ Galangin	44.82	265, 357	269.0454	269.0454	169.1 (12.64), 171.0 (10.87), 213.0 (10.73), 143.0 (8.90), 223.0 (8.03,) 195.0 (7.34)	C_15_H_9_O_5_	−0.2	−0.8	11.0
126	^B^ Isosakuranetin dihydrochalcone	44.91	291	287.0925	243.2789	166.1 (70.19), 152.1 (32.79), 119.1 (27.87), 188.2 (24.60), 203.2 (23.97), 186.2 (20.81), 122.1 (18.36), 228.2 (16.99), 125.1 (14.66), 287.2 (14.92), 254.2 (13.89), 201.21 (11.46), 135.1 (8.29), 269.2 (7.27), 107.2 (6.87), 213.2 (6.61), 161.2 (5.14), 138.2 (4.19), 146.2 (3.57)	C_16_H_15_O_5_	0.0	0.1	9.0
127	^A^ Pinocembrin dihydrochalcone	45.45	287	257.0820	213.2040	173.2 (66.35), 151.1 (33.34), 171.2 (31.95), 156.2 (24.29), 122.1 (19.76), 257.2 (12.86), 169.3 (13.48), 239.3 (11.24)	C_15_H_13_O_4_	−0.1	−0.4	9.0
128	^A^ Apigenin 3’-methyl ether (Acacetin) or ^A^ Apigenin 7-methyl ether (Genkwanin)	45.45	267, 338	283.0619	268.2004	240.2 (6.84), 151.1 (4.25)	C_16_H_11_O_5_	−0.7	−2.3	11.0
129	^B^ Caffeic acid pentyl or isopentyl ester	46.52	324	249.1138	161.1050	-	C_14_H_17_O_4_	−0.6	−2.3	6.0
130	^A^ Caffeic acid phenethyl ester (CAPE)	46.82	326	283.0981	135.1231	161.1 (46.24), 179.1 (20.40)	C_17_H_15_O_4_	−0.6	−2.0	10.0
131	^A^ Kaempferol 3’-methyl ether (Kaempferide)	46.89	267, 364	299.0564	165.1098	163.1 (76.38), 256.2 (73.45), 243.2 (69.45), 284.2 (70.50), 271.2 (64.61), 151.0 (53.68), 228.2 (49.64), 178.1 (39.93), 212.2 (32.76), 240.2 (23.93)	C_16_H_11_O_6_	−0.3	−0.9	11.0
132	^B^ Pinobanksin 3-acetate	47.27	295	313.0725	253.051	197.1 (5.86), 271.1 (5.36), 209.1 (4.75), 143.0 (3.17)	C_17_H_13_O_6_	−0.7	−2.3	16.0
133	^B^ Kaempferol methyl ether isomer II	47.52	264, 360	299.0561	284.2051	151.1 (32.52), 164.1 (9.83), 107.2 (6.51), 132.1 (5.38), 299.2 (3.39), 228.2 (3.31)	C_16_H_11_O_6_	0.1	0.2	11.0
134	^B^ Tetramethyl flavonoid	47.84	ND/-	329.0669	299.1782	271.2 (41.49), 314.2 (14.48)	C_17_H_13_O_7_	−0.2	−0.6	11.0
135	^B^ Methoxychrysin	47.87	265	283.0614	211.0405	239.0 (65.55), 268.0 (8.80)	C_16_H_11_O_5_	−0.2	−0.6	11.0
136	^C^ 2’,6’-Dihydroxy−4’-methoxypentanophenone	48.00	287	223.0983	152.0864	124.1 (77.51), 193.1 (13.04), 125.1 (11.95), 175.1 (6.65), 208.2 (5.84), 96.2 (6.22), 223.2 (3.86), 191.2 (3.24), 205.3 (2.47), 162.2 (2.47)	C_12_H_15_O_4_	−0.7	−3.0	5.0
137	Unidentified	48.12	310	219.1033	117.1531	145.1 (48.72), 119.1 (7.85)	C_13_H_15_O_3_	−0.7	−3.1	6.0
138	Unidentified	48.93	310	219.1028	117.3711	145.1 (32.70)	C_13_H_15_O_3_	−0.2	−0.8	6.0
139	^B^ Kaempferol 3,4’-dimethyl ether (Ermanin)	49.93	350, 267	313.0719	283.2122	255.2 (24.32), 253.2 (17.11), 298.2 (10.64)	C_17_H_13_O_6_	−0.1	−0.3	11.0
140	^B^*p*-Coumaric acid 3-methyl−3-butenyl ester	50.27	310	231.1028	117.1725	119.1 (90.59), 145.1 (49.02), 163.1 (4.99)	C_14_H_15_O_3_	−0.1	−0.4	7.0
141	^B^ 2-Acetyl−1,3-di-*p-*coumaroylglycerol	50.51	312	425.1242	163.0403	145.0 (53.67), 119.0 (49.02), 219.1 (11.88), 215.1 (6.36), 237.1 (5.21), 171.1 (5.05), 117.0 (4.31)	C_23_H_21_O_8_	0.0	0.1	13.0
142	^B^ 1-Acetyl−2-*p*-coumaroyl−3-feruloylglycerol	51.48	315	455.1347	163.1173	193.2 (78.06), 134.2 (46.98), 145.1 (41.86), 175.1 (42.27), 119.1 (40.73)	C_24_H_23_O_9_	0.1	0.2	13.0
143	^B^ 1-Acetyl−2,3-di-*p-*coumaroylglycerol	51.68	311	425.1244	163.1361	145.1 (64.46), 119.1 (57.20), 219.2 (13.02), 171.3 (7.70)	C_23_H_21_O_8_	−0.2	−0.4	13.0
144	^B^*p*-Coumaric acid 3-methyl−2-butenyl or 2-methyl−2-butenyl	51.75	311	231.1027	117.2347	-	C_14_H_15_O_3_	0.0	0.0	7.0
145	^B^*p*-Coumaric acid 3-methyl−2-butenyl or 2-methyl−2-butenyl	52.43	311	231.1029	117.2403	-	C_14_H_15_O_3_	−0.2	−0.9	7.0
146	Unidentified	53.16	ND/-	311.2237	157.1924	153.3 (41.78), 187.2 (5.50), 135.3 (5.35), 113.3 (4.75)	C_18_H_31_O_4_	−0.9	−3.0	3.0
147	^B^*p-*Coumaric acid benzyl ester	53.48	316	253.0869	117.2666	145.1 (12.89), 121.3 (3.15)	C_16_H_13_O_3_	0.1	0.3	10.0
148	Unidentified	54.60	299, 329	433.0921	243.2264	271.2 (41.07), 415.4 (26.05), 161.1 (19.62), 253.3 (11.06), 125.1 (7.62), 135.1 (6.88), 152.1 (5.62), 180.1 (4.85), 165.1 (4.69), 227.3 (4.97), 199.2 (3.56), 371.4 (3.43), 225.3 (3.13), 280.2 (2.54)	C_24_H_17_O_8_	0.8	1.7	16.0
149	^B^ Ferulic acid benzyl ester	54.92		283.0979	133.1788	147.3 (16.46), 119.2 (8.42)	C_17_H_15_O_4_	−0.3	−1.0	10.0
150	^B^ Caffeic acid phenylpropenyl ester	55.71	325	295.0978	134.1210	-	C_18_H_15_O_4_	−0.2	−0.7	11.0
151	^B^ Caffeic acid phenylpropyl ester	55.85	326	297.1139	161.1417	135.1 (44.14), 297.3 (15.52), 179.2 (11.00), 137.2 (4.01)	C_18_H_17_O_4_	−0.7	−2.2	10.0
152	^B^ Pinobanksin 3-propanoate	57.82	294	327.0878	253.2179	197.2 (5.41), 209.2 (3.72), 271.3 (2.71), 143.2 (2.09)	C_18_H_15_O_6_	−0.4	−1.2	11.0
153	^B^ Caffeic acid hexyl or isohexyl ester isomer I	57.99	ND/-	263.1298	134.5851	161.1 (73.06), 135.1 (51.49), 179.1 (10.43), 263.3 (10.02)	C_15_H_19_O_4_	−0.9	−3.3	6.0
154	^B^*p-*Coumaric acid phenethyl ester	58.08	310	267.1031	119.1235	145.1 (76.86), 117.2 (81.82), 163.1 (11.95)	C_17_H_15_O_3_	−0.5	−1.8	10.0
155	Unidentified	58.78	ND/-	233.1192	152.0855	124.1 (81.75)	C_14_H_17_O_3_	−0.8	−3.6	6.0
156	^B^ Caffeic acid hexyl or isohexyl ester isomer II	59.51	ND/-	263.1294	161.1533	135.1 (70.52), 263.3 (14.21)	C_15_H_19_O_4_	−0.5	−1.9	6.0
157	Unidentified	59.68	ND/-	403.1187	293.2895	109.1 (43.64), 171.2 (26.99), 189.1 (17.54), 255.3 (19.17), 189.2 (16.73), 385.4 (16.30), 403.4 (14.83), 265.4 (10.04), 187.2 (7.72), 211.2 (7.08), 213.2 (6.59), 251.2 (5.91), 145.2 (5.09), 317.3 (4.94), 249.3 (4.31), 231.2 (3.90), 359.4 (4.00), 202.2 (3.44), 299.6 (3.66)	C_24_H_19_O_6_	0.1	0.1	15.0
158	^B^ Pinostrobin chalcone	60.28	343	269.0827	122.0703	165.1 (83.49), 253.4 (86.88), 177.2 (49.29), 226.2 (47.58), 171.1 (35.51), 150.1 (31.31), 163.1 (21.30), 269.2 (16.42), 136.1 (13.47), 198.2 (14.25)	C_16_H_13_O_4_	−0.3	−0.8	10.0
159	Unidentified	61.08	ND/-	403.1194	281.2707	135.1 (32.65), 255.3 (34.47), 237.2 (29.91), 267.3 (26.49), 109.1 (17.43), 171.3 (14.49), 177.1 (12.64), 211.2 (12.58), 403.5 (10.85), 293.3 (7.11), 163.2 (4.33), 239.2 (3.69), 295.2 (3.70), 151.1 (3.50), 213.3 (3.82), 169.1 (2.89), 187.2 (2.60), 195.2 (2.49), 145.2 (2.40), 190.1 (2.25), 299.2 (2.19)	C_24_H_19_O_6_	−0.1	−1.7	15.0
160	^C^ 2’,6’-Dihydroxy−4’,4-dimethoxy dihydrochalcone (Calomelanone)	61.32	285	301.1091	152.1075	124.1 (55.29), 253.2 (54.38), 165.4 (23.22), 268.3 (20.76), 301.3 (12.14), 180.1 (8.43), 119.1 (7.49), 283.2 (8.06), 188.1 (6.14), 193.2 (6.40), 203.3 (3.19)	C_17_H_17_O_5_	−0.9	−3.1	9.0
161	^B^ 2’,6’-Dihydroxy−4’-methoxy dihydrochalcone	61.90	286	271.0979	152.0937	124.1 (60.13), 210.2 (27.77), 238.3 (25.34), 173.2 (13.05), 165.1 (10.13), 271.2 (7.97), 253.2 (6.31)	C_16_H_15_O_4_	−0.3	−1.1	9.0
162	^A^ Tectochrysin (Chrysin 7-methyl ether) [M+H]!	62.70	267, 310sh	269.0815	269.2764	226.2 (59.49), 254.2 (23.65), 167.1 (8.30), 270.5 (6.16), 186.3 (4.73), 129.1 (2.37), 209.2 (2.14)	C_16_H_13_O_4_	−0.7	−2.5	11.0
163	^B^ Pinobanksin 3-butenoate or isobutenoate	62.91	ND/-	339.0880	253.2128	197.2 (5.11), 209.1 (3.28)	C_19_H_15_O_6_	−0.5	−1.6	12.0
164	^A^ Pinostrobin (Pinocembrin 7-methyl ether) [M+H]!	63.20	289	271.0969	167.1288	131.1 (33.29), 103.2 (24.27), 269.3 (11.76), 226.3 (8.78), 271.2 (4.49), 270.5 (3.31), 254.3 (2.97), 186.3 (2.31), 165.2 (2.29)	C_16_H_15_O_4_	−0.4	−1.5	10.0
165	Unidentified	63.92	*351	551.1708	267.2518	283.2 (46.28), 255.3 (28.92), 551.6 (5.49), 281.2 (3.07), 135.1 (2.44), 429.5 (2.48)	C_33_H_27_O_8_	0.4	0.6	20.0
166	^B^*p-*Coumaric acid cinnamyl ester	63.94	313	279.1029	117.3253	-	C_18_H_15_O_3_	−0.3	−1.0	11.0
167	Unidentified	64.26	*310	281.1193	117.5723	145.2 (61.96), 121.1 (2.83), 281.3 (2.54)	C_18_H_17_O_3_	−0.1	−3.6	10.0
168	^B^ Caffeic acid heptyl or isoheptyl ester	64.41	ND/-	277.1453	161.1393	135.1 (62.26), 277.3 (19.86), 179.2 (12.63)	C_16_H_21_O_4_	−0.7	−2.6	6.0
169	^B^ Pinobanksin 3-butanoate or isobutanoate	64.74	293	341.1037	253.2173	197.2 (4.89), 209.2 (3.17)	C_19_H_17_O_6_	−0.6	−1.8	11.0
170	Unidentified	64.81	ND/-	387.1239	387.4150	171.2 (61.15), 173.1 (47.69), 283.2 (42.97), 197.2 (32.37), 343.8 (33.65), 215.2 (14.04), 255.2 (12.57), 301.4 (13.69), 211.3 (10.84), 169.2 (9.51), 239.4 (7.86), 145.2 (6.57), 156.2 (5.92), 281.2 (4.77), 359.4 (5.34), 183.3 (3.92), 147.2 (3.44), 226.3 (3.24), 213.2 (2.60), 259.3 (2.47)	C_24_H_19_O_5_	−0.2	−0.4	15.0
171	^C^ Balsacone A/B/E/F isomer I	65.06	266,289	419.1510	419.4067	375.4 (53.98), 283.3 (28.14), 257.2 (17.67), 173.2 (13.80), 389.4 (12.76), 203.5 (13.11), 213.2 (8.97), 298.3 (8.44), 401.3 (7.43), 152.1 (5.63), 254.2 (5.53), 311.3 (5.42), 171.2 (4.79), 333.4 (5.16)	C_25_H_23_O_6_	−1.0	−2.4	14.0
172	Unidentified	65.21	262, 347	387.1240	387.4117	281.2 (97.94), 267.2 (78.24), 171.2 (60.37), 119.1 (46.94), 283.3 (43.02), 237.2 (36.62), 173.2 (27.63), 197.3 (28.18), 177.1 (23.64), 343.4 (27.07), 293.4 (21.25), 252.4 (17.83), 163.1 (12.94), 255.2 (12.36), 145.2 (11.09), 169.2 (10.43), 156.2 (10.70), 148.3 (11.59), 211.2 (9.84), 239.2 (9.08), 301.4 (9.81)	C_24_H_19_O_5_	−0.2	−0.6	15.0
173	^C^ Balsacone A/B/E/F isomer II	65.37	266,289	419.1502	299.3067	313.3 (99.88), 419.5 (60.65), 119.1 (37.21), 375.4 (37.37), 178.2 (23.02), 269.4 (15.55), 325.4 (12.80), 203.2 (10.30), 152.1 (8.19), 192.2 (7.87), 137.1 (6.24), 213.6 (7.04), 254.4 (5.10), 285.4 (5.04), 257.3 (4.35), 93.1 (3.44), 145.2 (3.48), 265.3 (3.48), 173.2 (3.50), 287.3 (3.31), 243.2 (2.63), 295.3 (2.53), 163.2 (2.29)	C_25_H_23_O_6_	−0.2	−0.5	14.0
174	Unidentified	65.39	ND	469.1875	341.4908	469.5 (96.13), 257.2 (38.88), 357.4 (25.58), 383.8 (26.07), 311.3 (19.05), 438.4 (18.53), 328.3 (14.76), 339.3 (13.56), 327.8 (24.60), 125.1 (7.25), 297.3 (7.74), 215.2 (7.16), 242.2 (5.92), 223.3 (5.67), 353.3 (3.21)	C_26_H_29_O_8_	−0.7	−1.5	12.0
175	^B^ Pinobanksin 3-pentenoate or isopentenoate isomer I	65.40	292	353.1039	253.2231	197.2305 (4.88), 209.1898 (2.96)	C_20_H_17_O_6_	−0.9	−2.5	12.0
176	^C^ Balsacone C or Balsacone D	65.72	266,289	389.1402	283.3324	269.3 (90.93), 119.1 (47.02), 345.4 (58.63), 389.5 (54.23), 173.1 (25.16), 239.4 (23.64), 178.1 (17.40), 213.2 (13.48), 295.2 (12.39), 257.2 (9.36), 171.2 (9.05), 152.1 (9.06), 281.4 (11.13), 267.2 (7.85), 235.2 (7.28), 265.2 (7.13), 145.1 (6.59), 191.3 (6.98)	C_24_H_21_O_5_	−0.7	−1.9	14.0
177	Unidentified	65.74	290	469.1877	469.4952	437.5 (32.16), 343.3 (24.07), 353.4 (16.39), 341.3 (15.58), 223.3 (11.52), 385.4 (11.04), 325.4 (10.76), 393.5 (9.40), 257.3 (8.54), 297.4 (7.75), 215.3 (7.33), 357.4 (7.13), 311.6 (8.16), 125.1 (4.21), 280.6 (5.98), 189.2 (3.63), 367.4 (4.22)	C_26_H_29_O_8_	−0.9	−1.9	12.0
178	^B^ Pinobanksin 3-pentenoate or isopentenoate isomer II	65.74	282	353.1035	253.2266	271.2 (26.83), 197.3 (5.55), 209.6 (3.51), 225.3 (2.59)	C_20_H_17_O_6_	−0.5	−1.9	12.0
179	Unidentified	65.76	ND/-	387.1238	267.3253	119.1 (58.67), 281.2 (57.66), 177.2 (26.82), 387.8 (34.42), 163.1 (16.67), 293.2 (14.40), 283.2 (9.40), 239.2 (7.44), 345.3 (6.47), 237.3 (6.12), 173.2 (4.93), 225.2 (4.67), 255.7 (5.16), 197.2 (3.28)	C_24_H_19_O_5_	0.0	−0.1	15.0
180	Unidentified	66.01	ND/-	417.1336	297.2710	119.1 (73.94), 311.3 (69.24), 417.3 (40.26), 163.1 (20.53), 177.1 (20.29), 323.3 (12.23), 293.3 (8.90), 283.2 (7.45), 267.3 (6.89), 282.7 (12.85), 285.3 (2.95)	C_25_H_21_O_6_	0.8	1.9	15.0
181	Unidentified	66.10	ND/-	413.1972	134.2314	161.1 (98.90), 179.1 (23.69), 137.1 (11.08), 395.3 (6.95), 251.3 (7.21), 325.7 (4.12)	C_24_H_29_O_6_	−0.2	−0.5	10.0
182	Unidentified	66.24	ND/-	399.2180	134.1583	178.4 (38.54), 399.5 (21.28), 161.2 (4.22)	C_24_H_31_O_5_	−0.3	−0.7	9.0
183	Unidentified	66.57	ND/-	417.1349	135.1393	295.3 (28.85), 109.1 (20.68), 281.3 (13.63), 269.2 (11.44), 252.9 (5.46), 307.3 (2.47), 267.2 (2.40), 238.3 (2.36)	C_25_H_21_O_6_	−0.5	−1.2	15.0
184	Unidentified	66.60	ND/-	399.2176	134.2299	179.1 (15.44), 399.5 (14.91), 137.1 (10.24), 139.1 (2.82), 121.1 (2.64)	C_24_H_31_O_5_	0.1	0.3	9.0
185	^B^ Pinobanksin 3-benzoate	66.80	64.81	375.0878	253.2202	197.1 (4.84), 225.2 (3.56), 121.2 (3.04), 209.2 (2.85)	C_22_H_15_O_6_	−0.4	−1.0	15.0
186	Unidentified	67.12	ND/-	377.1396	258.2083	377.4 (78.69), 344.4 (17.20), 271.4 (13.77), 359.5 (12.78), 230.3 (12.47), 165.1 (8.77), 316.4 (9.27), 362.4 (8.55), 138.1 (4.54), 245.3 (5.43), 269.2 (3.55), 173.3 (2.37), 243.2 (2.03)	C_23_H_21_O_5_	−0.2	−0.5	13.0
187	^B^ Pinobanksin derivate	67.49	291	389.1037	253.2235	271.2 (48.46), 197.2 (5.15), 225.2 (3.00)	C_23_H_17_O_6_	−0.7	−1.7	15.0
188	Unidentified	67.63	ND	295.2290	277.4654	171.2 (70.40), 295.5 (10.03)	C_18_H_31_O_3_	−1.2	−4.0	3.0
189	^B^ Pinobanksin 3-pentanoate or isopentenoate isomer I	67.76	293	355.1192	253.2167	197.2 (4.62), 271.2 (3.55), 209.2 (2.17)	C_20_H_19_O_6_	−0.5	−1.5	11.0
190	^B^ Pinobanksin 3-pentanoate or isopentenoate isomer II	67.91	293	355.1194		197.2 (4.47), 209.2 (2.52)	C_20_H_19_O_6_	−0.6	−1.8	11.0
191	^C^ Caffeic acid monoterpene (geranyl) ester	68.07	326	315.1604	134.2007	137.1 (5.27), 179.2 (2.20), 106.1 (1.86)	C_19_H_23_O_4_	−0.2	−0.8	8.0
192	Unidentified	68.20	ND/-	401.1403	371.3482	401.4 (51.83), 297.2 (18.94), 267.2 (11.76), 385.6 (7.59), 254.3 (6.17), 171.2 (4.55), 295.2 (4.52), 282.3 (4.10), 197.2 (2.99), 226.4 (3.29), 343.2 (2.32)	C_25_H_21_O_5_	−0.8	−2.0	15.0
193	Unidentified	68.49	ND/-	403.1557	373.3656	403.4 (80.28), 269.2 (13.96), 385.9 (18.04), 297.4 (10.90), 370.5 (9.85), 355.3 (7.55), 342.4 (5.91), 271.3 (5.43), 173.2 (3.54), 309.3 (3.05), 241.2 (2.33)	C_25_H_23_O_5_	−0.6	−1.6	14.0
194	^B^ Pinobanksin 3-hexenoate or isohexenoate	68.54	ND/-	367.1189	253.2181	271.2 (31.89), 197.3 (5.77), 209.5 (3.20), 225.3 (2.91)	C_21_H_19_O_6_	−0.2	−0.4	12.0
195	Unidentified	68.70	ND/-	397.2018	145.1398	118.4 (56.33), 163.2 (26.66), 251.4 (16.67), 121.1 (4.44)	C_24_H_29_O_5_	0.2	0.6	10.0
196	^C^ Ricinoleic acid or 8-(3-octyloxiran−2-yl)octanoic acid	68.84	ND/-	297.2435	297.4100	171.2 (28.17)	C_18_H_33_O_3_	0.0	0.0	2.0
197	^C^ Balsacone L	68.86	*264, 344	519.1804	267.2076	269.2 (30.61), 519.8 (15.48), 399.5 (11.70), 251.3 (8.02), 471.7 (7.67), 413.3 (4.76), 119.1 (3.28), 279.2 (3.01), 293.6 (3.09)	C_33_H_27_O_6_	0.9	1.8	20.0
198	^B^ Pinobanksin 3-cinnamate	68.89	278	401.1033	253.2046	197.1602 (4.77), 225.2060 (2.94)	C_24_H_17_O_6_	−0.2	−0.6	16.0
199	Unidentified	69.01	ND/-	401.1390	119.1599	279.2 (73.08), 281.4 (29.74), 254.2 (23.23), 295.2 (21.52), 401.4 (22.55), 93.1 (8.97), 175.3 (9.77), 297.3 (9.24), 267.3 (8.38), 358.3 (7.40), 386.3 (7.06), 307.2 (5.82), 238.2 (4.23), 171.2 (3.75), 269.3 (3.18), 161.12 (2.92), 163.3 (3.07)	C_25_H_21_O_5_	0.4	1.1	15.0
200	^B^ Pinobanksin 3-hydroxycinnamate	69.16	285	403.1197	253.2276	271.2 (4.98), 197.2 (4.05), 225.3 (2.92), 149.1 (2.44)	C_24_H_19_O_6_	−1.0	−2.5	15.0
201	^C^ Balsacone J or Balsacone P	69.16	ND/-	521.1971	401.4243	521.6 (62.86), 415.4 (54.23), 119.1 (40.59), 295.3 (40.43), 281.2 (23.40), 307.3 (12.76), 309.3 (11.57), 269.2 (9.67), 389.4 (9.17), 399.4 (8.49), 283.2 (5.57), 427.5 (6.46), 519.5 (5.15), 321.4 (4.44), 477.4 (3.18), 345.3 (2.93), 267.4 (3.17)	C_33_H_29_O_6_	−0.1	−0.3	19.0
202	^B^ Metoxycinnamic acid cinnamyl ester isomer I	69.27	282	293.2125	293.4701	185.2 (57.87), 125.2 (49.45), 141.2 (18.74), 197.3 (15.90), 97.2 (11.61)	C_18_H_29_O_3_	−0.3	−0.9	4.0
203	Unidentified	69.29	*272	403.1557	119.1396	281.3 (83.80), 283.3 (38.72), 297.3 (35.80), 403.5 (18.39), 93.1 (12.52), 309.3 (11.57), 269.2 (8.11), 178.1 (7.02), 279.3 (6.06), 164.3 (6.49), 263.6 (6.04), 173.2 (3.81), 271.3 (2.39), 295.3 (2.31)		−0.6	−1.4	14.0
204	^B^ Pinobanksin 3-hexanoate or isohexanoate isomer I	69.57	294	369.1347	253.2138	271.2 (4.95), 197.2 (3.43), 225.1 (2.37), 115.2 (1.95)	C_21_H_21_O_6_	−0.3	−0.8	11.0
205	^B^ Pinobanksin 3-heptenoate or isoheptenoate isomer I	69.72	ND/-	381.1351	253.2257	197.2 (4.11), 271.3 (4.03), 225.2 (1.96)	C_22_H_21_O_6_	−0.7	−1.9	12.0
206	^B^ Metoxycinnamic acid cinnamyl ester isomer II	69.82	282	293.2120	293.3632	185.2 (59.65), 125.2 (51.93)	C_18_H_29_O_3_	0.3	0.9	4.0
207	^B^ Pinobanksin 3-hexanoate or isohexanoate isomer II	69.87	ND/-	369.1347	253.2245	197.2 (4.52), 271.2 (3.90), 225.3 (2.22), 209.2 (1.98), 115.2 (1.93)	C_21_H_21_O_6_	−0.3	−0.8	11.0
208	^C^ Iryantherin D or Balsacone K	70.20	ND/-	551.2078	299.2895	251.3 (21.30), 551.6 (22.85), 445.4 (7.98), 287.2 (5.17), 419.5 (4.72), 311.3 (4.26), 257.2 (2.60)	C_34_H_31_O_7_	0.2	0.3	19.0
209	Unidentified	70.26	ND/-	343.2855	283.3972	211.3 (96.37), 197.3 (72.36), 253.4 (30.83), 279. 5 (19.71)	C_20_H_39_O_4_	−0.1	−0.3	1.0
210	Unidentified	70.64	ND/-	295.2286	295.4295	141.2 (39.76), 125.2 (19.27)	C_18_H_31_O_3_	−0.7	−2.5	3.0
211	Unidentified	70.78	ND/-	489.3585	489.6854	427.6 (28.38), 445.6 (8.05), 471.6 (2.35)	C_30_H_49_O_5_	0.1	0.1	6.0
212	^B^ Pinobanksin 3-phenylpentenoate or phenylisopentenoate ester	70.97	*291	429.1344	253.2249	271.2 (57.79), 197.2 (3.17), 225.4 (3.81)	C_26_H_21_O_6_	0.0	−0.1	16.0
213	Unidentified	71.37	ND/-	505.3391	283.4780	-	C_26_H_49_O_9_	−0.9	−1.8	4.0
214	^C^ 2-Hydroxyethyl palmitate or 12-Hydroxystearic acid	71.52	ND/-	299.2595	299.4604	253.6 (14.77), 281.3 (7.78), 113.2 (6.13)	C_18_H_35_O_3_	−0.3	−1.0	1.0
215	Unidentified	71.84	ND/-	491.3590	311.5273	-	C_26_H_51_O_8_	−0.1	−0.1	1.0
216	Unidentified	72.43	ND/-	473.2337	473.5999	229.1 (15.75), 320.4 (17.64), 216.2 (8.35), 280.2 (5.94), 267.3 (3.38), 292.3 (2.68), 188.2 (2.50)	C_30_H_33_O_5_	−0.3	−0.6	14.0
217	Unidentified	72.48	ND/-	477.2644	255.2330	477.6 (4.09), 475.5 (3.45), 211.6 (3.10), 151.1 (1.97)	C_30_H_37_O_5_	0.2	0.5	12.0
218	Unidentified	73.07	ND/-	519.3542	297.4605	-	C_27_H_51_O_9_	−0.3	−0.6	2.0
219	Unidentified	73.85	ND/-	371.3171	311.4860	225.4 (63.83), 239.4 (61.36)	C_22_H_43_O_4_	−0.5	−1.3	1.0
220	Unidentified	74.37	ND/-	533.3707	311.5549	-	C_28_H_53_O_9_	−1.2	−2.2	2.0
221	Unidentified	74.70	ND/-	533.3703	311.3955	-	C_28_H_53_O_9_	−0.8	−1.5	2.0
222	Unidentified	76.33	ND/-	447.3329	-	-	C_24_H_47_O_7_	−0.2	−0.3	1.0
223	Unidentified	77.88	ND/-	561.4021	339.6137	211.3 (30.50)	C_30_H_57_O_9_	−1.3	−2.4	2.0

Abbreviations: **A(%)**—relative abundance; **RT**—retention time; **^A^**—identification by comparison of UV and MS/MS spectra with standards (the highest level of confidence); **^B^**—identification by comparison of MS/MS and/or UV spectrum with literature (good level of confidence); **^C^**—component was identified according to deprotonated molecular ion formula and prediction from MS/MS spectra detected in *Populus* genus in literature, but there are no sufficient MS and/or UV data (average/weak level of confidence); **^D^**—component was identified according to deprotonated molecular ion and prediction from MS spectra, but there are no sufficient MS/MS, and UV data and components have not been reported in poplars in literature (the weakest level of confidence); **[M + H]!**—components does not produce ions in ESI-negative mode; therefore, positive fragmentation was presented; **ND/-**—UV maximum was not determined due to low concentration, overlapping peaks or lack of UV absorption by components; *—UV maximum was evaluated approximately due to low concentration or overlapping peaks.

**Table 2:** Comparison of antimicrobial effect (MIC and MBC (μg/mL)) of ethanolic and ethanolic-water extracts of *Populus* buds.

Extract	*B. cereus*	*B. subtilis*	*E. faecalis*	*M. luteus*	*S. aureus*	*S. epidermidis*	*E. coli*	*K. pneumoniae*	*P. mirabilis*	*P. aeruginosa*	*S.* Typhimurium	*H. pylori*	*C. glabrata*	*C. albicans*	*C. parapsilosis*
**P.BA.EtOH**	**62.5/1000 ^S^**	125/250 ^C^	500/500 ^C^	**31.3/31.3 ^C^**	125/125 ^C^	125/250 ^C^	>1000/>1000	>1000/>1000 ^N^	>1000/>1000 ^N^	>1000/>1000	>1000/>1000 ^N^	**62.5/62.5 ^C^**	125/125 ^C^	125/250 ^C^	125/500 ^C^
**P.BA.W/E**	>1000/Nd ^N^	125/125 ^C^	125/>1000 ^N^	**62.5/62.5 ^C^**	125/125 ^C^	125/500 ^C^	>1000/Nd ^N^	>1000/Nd ^N^	>1000/Nd ^N^	>1000/N.d	>1000/Nd ^N^	125/>1000 ^N^	125/125 ^C^	125/125 ^C^	125/125 ^C^
P.CA.EtOH	125/>1000 ^N^	125/500 ^C^	250/1000 ^C^	125/125 ^C^	125/500 ^C^	125/500 ^C^	>1000/>1000 ^N^	>1000/>1000 ^N^	>1000/>1000 ^N^	>1000/>1000	>1000/>1000 ^N^	**62.5/62.5 ^C^**	125/250 ^C^	250/500 ^C^	**62.5/250 ^C^**
P.CA.W/E	1000/1000 ^C^	250/500 ^C^	500/1000 ^C^	125/250 ^C^	125/250 ^C^	250/250 ^C^	>1000/N.d ^N^	>1000/Nd ^N^	>1000/Nd ^N^	>1000/Nd	>1000/N.d ^N^	500/>1000 ^N^	250/250 ^C^	250/250 ^C^	250/500 ^C^
P.DE.EtOH	500/>1000 ^N^	125/125 ^C^	500/>1000 ^N^	125/125 ^C^	125/250 ^C^	250/1000 ^C^	>1000/>1000 ^N^	>1000/>1000 ^N^	>1000/>1000 ^N^	>1000/>1000	>1000/>1000 ^N^	**62.5/62.5 ^C^**	125/250 ^C^	250/250 ^C^	250/500 ^C^
**P.DE.W/E**	>1000/Nd ^N^	125/125 ^C^	500/1000 ^C^	**62.5/250 ^C^**	125/250 ^C^	500/500 ^C^	>1000/Nd ^N^	>1000/Nd ^N^	>1000/Nd ^N^	>1000/Nd	>1000/Nd ^N^	**62.5/62.5 ^C^**	250/250 ^C^	250/500 ^C^	250/500 ^C^
**P.DE** × **P.N.EtOH**	125/>1000 ^N^	125/500 ^C^	500/500 ^C^	125/125 ^C^	125/250 ^C^	250/500 ^C^	>1000/>1000 ^N^	>1000/>1000 ^N^	>1000/>1000 ^N^	>1000/>1000	>1000/>1000 ^N^	**62.5/62.5 ^C^**	125/500 ^C^	250/250 ^C^	125/250 ^C^
**P.DE** × **P.N.W/E**	>1000/Nd ^N^	125/125 ^C^	500/1000 ^C^	**62.5/250 ^C^**	125/250 ^C^	500/500 ^C^	>1000/Nd ^N^	>1000/Nd ^N^	>1000/Nd ^N^	>1000/Nd	>1000/Nd ^N^	500/>1000 ^N^	250/250 ^C^	250/500 ^C^	250/500 ^C^
**P.ER.EtOH**	**31.3/>1000 ^N^**	**62.5/125 ^C^**	250/500 ^C^	**62.5/125 ^C^**	125/125 ^C^	125/125 ^C^	>1000/>1000 ^N^	>1000/>1000 ^N^	>1000/>1000 ^N^	>1000/>1000	>1000/>1000 ^N^	**62.5/62.5 ^C^**	125/250 ^C^	250/250 ^C^	250/500 ^C^
**P.ER.W/E**	>1000/Nd ^N^	**62.5/125 ^C^**	250/>1000 ^N^	**62.5/125 ^C^**	125/125 ^C^	125/500 ^C^	>1000/Nd ^N^	>1000/Nd ^N^	>1000/Nd ^N^	>1000/Nd	>1000/Nd ^N^	250/>1000 ^N^	125/125 ^C^	250/250 ^C^	250/250 ^C^
**P.** × **KOM.EtOH**	**62.5/>1000 ^N^**	**62.5/125 ^C^**	125/250 ^C^	**62.5/62.5 ^C^**	**62.5/125 ^C^**	125/125 ^C^	>1000/>1000 ^N^	>1000/>1000 ^N^	>1000/>1000 ^N^	>1000/>1000	>1000/>1000 ^N^	**62.5/62.5 ^C^**	125/125 ^C^	125/250 ^C^	125/500 ^C^
**P.** × **KOM.W/E**	125/>1000 ^N^	125/125 ^C^	250/250 ^C^	**62.5/62.5 ^C^**	**62.5/125 ^C^**	125/250 ^C^	>1000/Nd ^N^	>1000/Nd ^N^	>1000/Nd ^N^	>1000/Nd	>1000/Nd ^N^	250/250 ^C C^	125/250 ^C^	250/250 ^C^	125/250 ^C^
**P.LAU.EtOH**	**62.5/>1000 ^N^**	125/125 ^C^	500/>1000 ^N^	125/125 ^C^	125/250 ^C^	250/>1000 ^S^	>1000/>1000 ^N^	>1000/>1000 ^N^	>1000/>1000 ^N^	>1000/>1000	>1000/>1000 ^N^	**62.5/62.5 ^C^**	250/250 ^C^	250/1000 ^C^	250/1000 ^C^
P.LAU.W/E	>1000/Nd ^N^	500/1000 ^C^	1000/>1000 ^N^	250/500 ^C^	500/1000 ^C^	1000/>1000 ^N^	>1000/Nd ^N^	>1000/Nd ^N^	>1000/Nd ^N^	>1000/Nd	>1000/Nd ^N^	250/1000 ^C^	>1000/Nd ^N^	>1000/Nd ^N^	>1000/Nd ^N^
P.LAS.EtOH	500/>1000 ^N^	250/1000 ^C^	1000/>1000 ^N^	250/250 ^C^	250/1000 ^C^	500/>1000 ^N^	>1000/>1000 ^N^	>1000/>1000 ^N^	>1000/>1000 ^N^	>1000/>1000	>1000/>1000 ^N^	250/500 ^C^	>1000/>1000 ^N^	>1000/>1000 ^N^	>1000/>1000 ^N^
P.LAS.W/E	>1000/Nd ^N^	500/1000 ^C^	1000/>1000 ^N^	250/500 ^C^	500/1000 ^C^	1000>1000 ^N^	>1000/Nd ^N^	>1000/Nd ^N^	>1000/Nd ^N^	>1000/Nd ^N^	>1000/Nd ^N^	>1000/Nd ^N^	>1000/Nd ^N^	>1000/Nd ^N^	>1000/Nd ^N^
**P.M.HB.ETOH**	**31.3/>1000 ^N^**	**62.5/125 ^C^**	125/250 ^C^	**31.3/31.3 ^C^**	**62.5/125 ^C^**	125/250 ^C^	>1000/>1000 ^N^	>1000/>1000 ^N^	>1000/>1000 ^N^	>1000/>1000	>1000/>1000 ^N^	**31.3/31.3 ^C^**	125/125 ^C^	125/125 ^C^	125/250 ^C^
**P.M.HB.W/E**	**62.5/1000 ^S^**	**62.5/62.5 ^C^**	250/250 ^C^	**62.5/62.5 ^C^**	**62.5/250 ^C^**	125/125 ^C^	>1000/Nd ^N^	>1000/Nd ^N^	>1000/Nd ^N^	>1000/Nd	>1000/Nd ^N^	250/250 ^C^	250/250 ^C^	250/250 ^C^	125/250 ^C^
**P.M** × **P.B.ETOH**	125/>1000 ^N^	**62.5/250 ^C^**	250/500 ^C^	**62.5/62.5 ^C^**	125/250 ^C^	125/250 ^C^	>1000/>1000 ^N^	>1000/>1000 ^N^	>1000/>1000 ^N^	>1000/>1000	>1000/>1000 ^N^	**31.3/31.3 ^C^**	125/125 ^C^	125/125 ^C^	125/250 ^C^
**P.M** × **P.B.W/E**	1000/>1000 ^N^	125/125 ^C^	250/1000 ^C^	**62.5/250 ^C^**	**62.5/125 ^C^**	125/250 ^C^	>1000/Nd ^N^	>1000/Nd ^N^	>1000/Nd ^N^	>1000/Nd	>1000/Nd ^N^	250/1000 ^C^	125/125 ^C^	125/125 ^C^	125/125 ^C^
**P.M** × **P.TRI.ETOH**	**62.5/1000 ^S^**	**62.5/125 ^C^**	**31.3/250 ^S^**	**15.6/15.6 ^C^**	**62.5/62.5 ^C^**	**15.6/62.5 ^C^**	>1000/>1000 ^N^	>1000/>1000 ^N^	>1000/>1000 ^N^	>1000/>1000	>1000/>1000 ^N^	**62.5/62.5 ^C^**	125/500 ^C^	125/1000 ^S^	125/500 ^C^
**P.M** × **P.TRI.W/E**	**31.3/>1000 ^N^**	**62.5/125 ^C^**	**62.5/125 ^C^**	**15.6/15.6 ^C^**	**62.5/62.5 ^C^**	**62.5/62.5 ^C^**	>1000/Nd ^N^	>1000/Nd ^N^	>1000/Nd ^N^	>1000/Nd	>1000/Nd ^N^	**62.5/125 ^C^**	125/125 ^C^	125/125 ^C^	125/250 ^C^
**P.N.1.ETOH**	**62.5/250 ^C^**	125/125 ^C^	125/250 ^C^	**31.3/31.3 ^C^**	125/250 ^C^	125/125 ^C^	>1000/>1000 ^N^	>1000/>1000 ^N^	>1000/>1000 ^N^	>1000/>1000	>1000/>1000 ^N^	**62.5/62.5 ^C^**	125/125 ^C^	125/250 ^C^	125/500 ^C^
P.N.1.W/E	>1000/Nd ^N^	250/500 ^C^	500/>1000 ^N^	125/500 ^C^	250/500 ^C^	500/1000 ^C^	>1000/Nd ^N^	>1000/Nd ^N^	>1000/Nd ^N^	>1000/Nd	>1000/Nd ^N^	125/250 ^C^	250/250 ^C^	500/500 ^C^	250/500 ^C^
**P.N.2.ETOH**	125/>1000 ^N^	125/125 ^C^	250/500 ^C^	**62.5/125 ^C^**	125/250 ^C^	250/500 ^C^	>1000/>1000 ^N^	>1000/>1000 ^N^	>1000/>1000 ^N^	>1000/>1000	>1000/>1000 ^N^	**62.5/125 ^C^**	125/125 ^C^	125/250 ^C^	**62.5/500 ^S^**
**P.N.2.W/E**	>1000/Nd ^N^	125/125 ^C^	125/250 ^C^	**62.5/62.5 ^C^**	125/125 ^C^	250/250 ^C^	>1000/Nd ^N^	>1000/Nd ^N^	>1000/Nd ^N^	>1000/Nd	>1000/Nd ^N^	125/250 ^C^	125/125 ^C^	125/250 ^C^	125/250 ^C^
**P.N.3.EtOH**	**31.3/62.5 ^C^**	**31.3/62.5 ^C^**	125/250 ^C^	**31.3/62.5 ^C^**	**62.5/62.5 ^C^**	**62.5/125 ^C^**	>1000/>1000 ^N^	>1000/>1000 ^N^	>1000/>1000 ^N^	>1000/>1000	>1000/>1000 ^N^	**31.3/31.3 ^C^**	**62.5/125 ^C^**	125/125 ^C^	125/250 ^C^
**P.N.3.W/E**	>1000/Nd ^N^	**62.5/62.5 ^C^**	250/250 ^C^	**62.5/62.5 ^C^**	**62.5/62.5 ^C^**	**62.5/125 ^C^**	>1000/Nd ^N^	>1000/Nd ^N^	>1000/Nd ^N^	>1000/Nd	>1000/Nd ^N^	250/250 ^C^	**62.5/125 ^C^**	125/125 ^C^	125/125 ^C^
**P.** × **PE1.EtOH**	**62.5/>1000 ^N^**	**62.5/250 ^C^**	125/250 ^C^	**31.3/62.5 ^C^**	**62.5/125 ^C^**	125/125 ^C^	>1000/>1000 ^N^	>1000/>1000 ^N^	>1000/>1000 ^N^	>1000/>1000	>1000/>1000 ^N^	**62.5/62.5 ^C^**	125/125 ^C^	125/125 ^C^	**62.5/250 ^C^**
**P.** × **PE1.W/E**	>1000/Nd ^N^	125/125 ^C^	250/250 ^C^	**31.3/62.5 ^C^**	**62.5/125 ^C^**	125/250 ^C^	>1000/Nd ^N^	>1000/Nd ^N^	>1000/Nd ^N^	>1000/Nd	>1000/Nd ^N^	250/250 ^C^	125/125 ^C^	125/125 ^C^	125/250 ^C^
P. × PE2.EtOH	125/>1000 ^N^	125/125 ^C^	500/1000 ^C^	125/125 ^C^	125/250 ^C^	250/1000 ^C^	>1000/>1000 ^N^	>1000/>1000 ^N^	>1000/>1000 ^N^	>1000/>1000	>1000/>1000 ^N^	**62.5/62.5 ^C^**	125/250 ^C^	250/500 ^C^	250/500 ^C^
P. × PE2.W/E	>1000/Nd ^N^	500/>1000 ^N^	1000/>1000 ^N^	125/500 ^C^	250/1000 ^C^	250/500 ^C^	>1000/Nd ^N^	>1000/Nd ^N^	>1000/Nd ^N^	>1000/Nd	>1000/Nd ^N^	1000/>1000 ^N^	500/500 ^C^	500/1000 ^C^	500/1000 ^C^
**P.** × **RA.EtOH**	**62.5/>1000 ^N^**	125/125 ^C^	500/500 ^C^	**31.3/62.5 ^C^**	125/250 ^C^	125/250 ^C^	>1000/>1000 ^N^	>1000/>1000 ^N^	>1000/>1000 ^N^	>1000/>1000	>1000/>1000 ^N^	**62.5/62.5 ^C^**	125/500 ^C^	250/500 ^C^	125/125 ^C^
**P.** × **RA.W/E**	125/>1000 ^N^	125/125 ^C^	500/500 ^C^	**62.5/62.5 ^C^**	125/125 ^C^	250/250 ^C^	>1000/Nd ^N^	>1000/Nd ^N^	>1000/Nd ^N^	>1000/Nd	>1000/Nd ^N^	500/500 ^C^	250/250 ^C^	250/250 ^C^	125/250 ^C^
**P.RO.EtOH**	**62.5/>1000 ^N^**	**31.3/62.5 ^C^**	**62.5/250 ^C^**	**15.6/31.3 ^C^**	**62.5/62.5 ^C^**	**15.6/31.3 ^C^**	>1000/>1000 ^N^	>1000/>1000 ^N^	>1000/>1000 ^N^	>1000/>1000	>1000/>1000 ^N^	**31.3/31.3 ^C^**	**62.5/62.5 ^C^**	125/125 ^C^	**62.5/250 ^C^**
**P.RO.W/E**	>1000/Nd ^N^	**31.3/62.5 ^C^**	**62.5/125 ^C^**	**15.6/31.3 ^C^**	**31.3/62.5 ^C^**	**31.3/31.3 ^C^**	>1000/Nd ^N^	>1000/Nd ^N^	>1000/Nd ^N^	>1000/Nd	>1000/Nd ^N^	**62.5/125 ^C^**	**62.5/250 ^C^**	125/125 ^C^	**62.5/125 ^C^**
**P.SI.EtOH**	**62.5/>1000 ^N^**	125/250 ^C^	250/500 ^C^	**62.5/125 ^C^**	125/250 ^C^	125/125 ^C^	>1000/>1000 ^N^	>1000/>1000 ^N^	1000/>1000 ^N^	>1000/>1000	>1000/>1000 ^N^	**62.5/62.5 ^C^**	125/125 ^C^	125/250 ^C^	125/250 ^C^
**P.SI.W/E**	125/>1000 ^N^	125/125 ^C^	250/500 ^C^	**62.5/250 ^C^**	125/250 ^C^	125/500 ^C^	>1000/Nd ^N^	>1000/Nd ^N^	>1000/Nd ^N^	>1000/Nd	>1000/Nd ^N^	250/500 ^C^	125/250 ^C^	250/250 ^C^	250/250 ^C^
**P.SU.EtOH**	**62.5/>1000 ^N^**	**62.5/125 ^C^**	250/250 ^C^	**31.3/62.5 ^C^**	**62.5/125 ^C^**	125/125 ^C^	>1000/>1000 ^N^	>1000/>1000 ^N^	>1000/>1000 ^N^	>1000/>1000	>1000/>1000 ^N^	**31.3/62.5 ^C^**	125/125 ^C^	125/250 ^C^	125/250 ^C^
**P.SU.W/E**	125/>1000 ^N^	125/125 ^C^	250/500 ^C^	**62.5/62.5 ^C^**	125/125 ^C^	125/250 ^C^	>1000/Nd ^N^	>1000/Nd ^N^	>1000/Nd ^N^	>1000/Nd	>1000/Nd ^N^	**62.5/62.5 ^C^**	125/125 ^C^	125/125 ^C^	125/250 ^C^
**P.TA.1.EtOH**	**62.5/500 ^S^**	125/125 ^C^	500/500 ^C^	**31.3/62.5 ^C^**	125/125 ^C^	125/250 ^C^	>1000/>1000 ^N^	>1000/>1000 ^N^	>1000/>1000 ^N^	>1000/>1000	>1000/>1000 ^N^	**62.5/62.5 ^C^**	125/125 ^C^	125/250 ^C^	125/500 ^C^
**P.TA.1.W/E**	>1000/Nd ^N^	125/125 ^C^	250/>1000 ^N^	**62.5/62.5 ^C^**	125/250 ^C^	125/500 ^C^	>1000/Nd ^N^	>1000/Nd ^N^	>1000/Nd ^N^	>1000/Nd	>1000/Nd ^N^	250/>1000 ^N^	125/250 ^C^	125/250 ^C^	125/250 ^C^
**P.TA.2.EtOH**	250/>1000 ^N^	125/125 ^C^	**62.5/500 ^S^**	**31.3/62.5 ^C^**	125/250 ^C^	125/250 ^C^	>1000/>1000 ^N^	>1000/>1000 ^N^	>1000/>1000 ^N^	>1000/>1000	>1000/>1000 ^N^	**62.5/62.5 ^C^**	125/125 ^C^	125/250 ^C^	125/250 ^C^
P.TA.2.W/E	>1000/Nd ^N^	250/250 ^C^	500/>1000 ^N^	125/500 ^C^	250/500 ^C^	250/500 ^C^	>1000/Nd ^N^	>1000/Nd ^N^	>1000/Nd ^N^	>1000/Nd	>1000/Nd ^N^	500/>1000 ^N^	250/250 ^C^	250/500 ^C^	500/500 ^C^
**P.TA1** × **PTRI.EtOH**	**15.6/1000 ^S^**	**62.5/62.5 ^C^**	125/250 ^C^	**15.6/15.6 ^C^**	**62.5/125 ^C^**	**31.3/62.5 ^C^**	>1000/>1000 ^N^	>1000/>1000 ^N^	>1000/>1000 ^N^	>1000/>1000	>1000/>1000 ^N^	**62.5/62.5 ^C^**	125/125 ^C^	125/500 ^C^	125/1000 ^S^
**P.TA1** × **PTRI.W/E**	>1000/Nd ^N^	**62.5/125 ^C^**	125/250 ^C^	**15.6/31.3 ^C^**	125/125 ^C^	**62.5/62.5 ^C^**	>1000/Nd ^N^	>1000/Nd ^N^	>1000/Nd ^N^	>1000/Nd	>1000/Nd ^N^	125/250 ^C^	125/250 ^C^	250/250 ^C^	250/250 ^C^
**P.TA.2** × **P.TRI.EtOH**	**31.3/500** ^S^	**31.3/31.3 ^C^**	**15.6/500 ^S^**	**7.8/7.8 ^C^**	**31.3/31.3 ^C^**	**7.8/15.6**	>1000/>1000 ^N^	>1000/>1000 ^N^	>1000/>1000 ^N^	>1000/>1000	>1000/>1000 ^N^	**31.3/31.3 ^C^**	125/125 ^C^	125/500 ^C^	1000/1000 ^C^
**P.TA.2** × **P.TRI.W/E**	**31.3/500** ^S^	**62.5/62.5 ^C^**	**62.5/125 ^C^**	**31.3/31.3 ^C^**	**62.5/62.5 ^C^**	31.3/125	>1000/Nd ^N^	>1000/Nd ^N^	>1000/Nd ^N^	>1000/Nd	>1000/Nd ^N^	**62.5/125 ^C^**	125/500 ^C^	125/500 ^C^	125/500 ^C^
**P.TRI.EtOH**	**31.3/>1000 ^N^**	**62.5/62.5 ^C^**	**62.5/125 ^C^**	**15.6/15.6 ^C^**	**62.5/62.5 ^C^**	**31.3/31.3 ^C^**	>1000/>1000 ^N^	>1000/>1000 ^N^	>1000/>1000 ^N^	>1000/>1000	>1000/>1000 ^N^	**31.3/31.3 ^C^**	**62.5/125 ^C^**	125/250 ^C^	125/500 ^C^
**P.TRI.W/E**	>1000/Nd ^N^	**62.5/62.5 ^C^**	**62.5/125 ^C^**	**15.6/31.3 ^C^**	**62.5/62.5 ^C^**	**31.3/500**	>1000/Nd ^N^	>1000/Nd ^N^	>1000/Nd ^N^	>1000/Nd ^N^	>1000/Nd ^N^	**62.5/125 ^C^**	**62.5/62.5 ^C^**	125/250 ^C^	**62.5/250 ^C^**
P. × WCA.EtOH	>1000/>1000 ^N^	>1000/>1000 ^N^	>1000/>1000 ^N^	1000/1000 ^C^	1000/1000 ^C^	1000/>1000 ^N^	>1000/>1000 ^N^	>1000/>1000 ^N^	>1000/>1000 ^N^	>1000/>1000 ^N^	>1000/>1000 ^N^	250/250 ^C^	>1000/>1000 ^N^	>1000/>1000 ^N^	>1000/>1000 ^N^
P. × WCA.W/E	>1000/Nd ^N^	>1000/Nd ^N^	>1000/Nd ^N^	1000/Nd ^N^	>1000/Nd ^N^	>1000/Nd ^N^	>1000/Nd ^N^	>1000/Nd ^N^	>1000/Nd ^N^	>1000/Nd ^N^	>1000/Nd ^N^	500/1000 ^C^	>1000/Nd ^N^	>1000/Nd ^N^	>1000/Nd ^N^
P.WIL.EtOH	>1000/>1000 ^N^	1000/>1000 ^N^	>1000/>1000 ^N^	500/1000 ^C^	1000/1000 ^C^	250/1000 ^C^	>1000/>1000 ^N^	>1000/>1000 ^N^	>1000/>1000 ^N^	>1000/>1000 ^N^	>1000/>1000 ^N^	250/250 ^C^	>1000/>1000 ^N^	>1000/>1000 ^N^	>1000/>1000 ^N^
P.WIL.W/E	>1000/>1000 ^N^	1000/>1000 ^N^	1000/>1000 ^N^	500/>1000 ^N^	1000/>1000 ^N^	1000/>1000 ^N^	>1000/Nd ^N^	>1000/Nd ^N^	>1000/Nd ^N^	>1000/Nd ^N^	>1000/Nd ^N^	1000/>1000 ^N^	>1000/Nd ^N^	1000/Nd ^N^	>1000/Nd ^N^
**Reference drugs**	0.98 ^VAN^	0.24 ^VAN^	1.95 ^VAN^	0.12 ^VAN^	0.98 ^VAN^	0.98 ^VAN^	0.015 ^CIP^	0.12 ^CIP^	0.03 ^CIP^	0.49 ^CIP^	0.06 ^CIP^	31.3 ^MET^	0.24 ^NYS^	0.48 ^NYS^	0.24 ^NYS^

Activity abbreviations: **^S^**—bacteriostatic or fungistatic effect; **^C^**—bactericidal or fungicidal effect; **^N^**—MBC/MIC or MFC/MIC ratio was not determined; **Nd**—MBC or MFC was not determined. Highly active samples as well as the lowest MICs are highlighted in bold (average, ≤62.5 μg/mL) or frame (high, ≤15.6 μg/mL). Poplar taxons acronyms: **P.BA**—*P*. *balsamifera;* **P.CA**—*P*. *cathayana*; **P.DE**—*P*. *deltoides*; **P.DE** × **P.N**—*P*. *deltoides* × *P*. *nigra*; **P.ER**—*P*. ‘Eridano’; **P. × KOM**—*P*. × *komarowii*; **P.LAU**—*P*. *laurifolia*; **P.LAS**—*P*. *lasiocarpa*; **P.MAX**—*P*. *maximowiczii*; **P.M** × **P.B**—*P*. *maximowiczii* × *P*. *berolinensis*; **P.M** × **P.TRI**—*P*. *maximowiczii* × *P*. *trichocarpa;* **P.N**—*P*. *nigra* (samples 1–3); **P. × PE**—*P*. × *petrowskiana* (samples 1–2); **P. × RA**—*P*. × *rasumoskowiana*; **P.RO**—*P*. *trichocarpa* ‘Rochester’; **P.SI**—*P*. *simonii*; **P.SU**—*P*. *suaveolens*; **P.TA**—*P*. *tacamahaca* (samples 1–2); **P.TA × P.TRI**—*P*. *tacamahaca* × *P*. *trichocarpa* (samples 1–2); **P.TRI**—*P*. *trichocarpa*, **P.WIL**—*P*. *wilsonii*, **P. × WCA**—*P*. × *wilsocarpa*. Reference drugs: **CIP**—ciprofloxacin; **MET**—metronidazole; **NYS**—nystatin; **VAN**—vancomycin.

## Data Availability

The data presented in this study are available on request from the corresponding author.

## Supplementary Materials

The following supporting information can be downloaded at: https://www.mdpi.com/article/10.3390/molecules29020437/s1, Table S1. Relative abundance of extract components and bud extraction yield.
