# Supplementary material for: Phytochemical Profiles and Antimicrobial Activity of Selected Populus spp. Bud Extracts
Source: Molecules. 2024 Jan 16;29(2):437. doi: 10.3390/molecules29020437 (PMC10819791; doi:10.3390/molecules29020437)
Supplement: Supplementary file 1 [file molecules-29-00437-s001.zip › molecules-2767293-supplementary.pdf]

Supplementary Table S1. Relative abundance of extracts components and buds extraction yield.

No.	Component	RT [min]
1	Unidentified	0.84
2	Unidentified	0.87
3	Unidentified	0.89
4	Unidentified	1.22
5	Leonuriside A	4.31
6	Chlorogenic acid	6.55
7	Caffeoylglucose isomer I	8.24
8	Caffeoylglucose isomer II	9.19
9	Vanilline	9.36
10	Salicyl alcohol dihexoside	10.02
11	Caffeoylglucose isomer III	10.24
12	*p*-Coumaric acid hexoside isomer I	10.54
13	Catechin or Epicatechin	10.87
14	Caffeic acid	11.46
15	di-Caffeoylglycerol	11.91
16	Feruloyl or isoferuloyl hexoside isomer I	12.14
17	Methoxybenzaldehyde	12.64
18	Caffeoylglycerol	13.06
19	*p*-Coumaric acid hexoside isomer II	13.11
20	Feruloyl or isoferuloyl hexoside isomer II	13.36
21	*p*-Coumaric acid	14.42
22	3,4,5-Trimethoxycinnamic acid	14.42
23	Salicortin	14.93
24	7-*O*-caffeoylsalirepin	15.18
25	Ferulic acid	15.20
26	Caffeic acid dihydroxypentyl or isopentyl ester isomer I	15.22
27	Isoferulic acid	15.71
28	Taxifolin (Dihydroquercetin) isomer I	16.00
29	Caffeic acid derivate	16.01
30	Caffeic acid derivate	16.23
31	Caffeic acid derivate	16.46
32	Caffeic acid derivate	16.65
33	Unidentified	16.67
34	Populoside isomer I	16.68
35	Azelaic acid (Nonanedioic acid)	17.07
36	Coumaric derivate	17.13
37	Populoside isomer II	17.33
38	Eriodictyol (Dihydroluteolin) isomer	17.37
39	Unidentified	17.40
40	Pinobanksin- or Naringenin-7-*O*-hexoside	17.57
41	Unidentified	17.69
42	Vanilloyl-methyl-ketone	17.79
43	Taxifolin (Dihydroquercetin) isomer II	17.84
44	Salireposide	18.27
45	Unidentified	18.46
46	Unidentified	18.48
47	Unidentified	18.48
48	Eriodictyol (Dihydroluteolin)	18.83
49	Isograndidentatin A	18.88
50	Grandidentatin	19.30
51	Caffeic acid dimethyl ether	19.48
52	Caffeic acid derivate	19.72
53	Dihydroisorhamnetin	20.29
54	Populoside isomer III	20.91
55	Apigenin-7-*O*-glucoside (Apigetrin)	21.10
56	Diosmetin 7-*O*-rutinoside	21.21
57	Unidentified	22.15
58	Caffeic acid derivate	22.22
59	Caffeic ethyl ester	22.65
60	Aromadendrin (Dihydrokaempferol)	23.08
61	Pinobanksin 5-methyl ether	23.40
62	Kaempferol 3-methyl ether (Isokaempferide)	23.66
63	Unidentified	23.83
64	di-Caffeoylglycerol	24.58
65	Quercetin	25.10
66	Caffeic acid derivate	25.33
67	Luteolin	25.40
68	Quercetin 3-methyl ether	26.85
69	Pinobanksin (Dihydrogalangin)	27.25
70	Boropinic acid (Caffeic acid 3-methyl, 4-(3-methyl-2-buten-1-yl) ether)	28.52
71	Naringenin (Dihydroapigenin)	28.60
72	Chrysin 5-methyl ether	28.84
73	Eriodictyol 3'-methyl ether (Homoeriodictyol) or Eriodictyol 4'-methyl ether (Hesperetin)	28.87
74	1-Caffeoyl-3-*p*-coumaroylglycerol	28.96
75	Flavonoid	29.55
76	Caffeic acid derivate	29.63
77	Caffeic acid propyl or isopropyl ester	29.88
78	Aromadendrin 7-methyl ether	30.36
79	Naringenin chalcone	30.45
80	Apigenin	30.51
81	Kaempferol	31.19
82	Caffeic acid hydroxyphenylethyl ester	31.47
83	Quercetin 3'-methyl ether (Isorhamnetin)	31.77
84	Quercetin methyl ether isomer I	32.28
85	Luteolin 5-methyl ether	33.03
86	Syringenin (sinapyl alcohol)	33.24
87	Caffeic acid butenyl or isobutenyl ester	33.73
88	Quercetin dimethyl ether isomer I	33.74
89	*p*-Coumaric acid derivate	33.81
90	1,3-di-*p*-Coumaroylglycerol	33.98
91	Galangin 5-methyl ether	34.41
92	1,2-di-*p*-Coumaroylglycerol	34.46
93	Pinobanksin 5-methyl ether 3-acetate	34.69
94	*m*-Coumaric acid (3-Hydroxycinnamic acid)	35.01
95	Pinobanksin 3-hydroxybutanoate isomer I	35.14
96	2-Acetyl-1,3-di-caffeoylglycerol	35.23
97	1-Acetyl-2,3-di-caffeoylglycerol	35.73
98	Caffeic acid derivate	35.81
99	Quercetin methyl ether isomer II	36.15
100	Quercetin 7-methyl ether (Rhamnetin)	36.53
101	Kaempferol methyl ether isomer I	36.68
102	Caffeic acid butyl or isobutyl ester isomer I	37.33
103	Pinobanksin 3-hydroxybutanoate isomer II	37.55
104	Unidentified	37.74
105	Caffeic acid butyl or isobutyl ester isomer II	37.96
106	2',4',6'-Trihydroxypentanophenone	38.89
107	Quercetin dimethyl ether isomer II	39.00
108	Caffeic acid 2-methyl-2-butenyl ester	39.19
109	Quercetin dimethyl ether isomer III	39.41
110	Caffeic acid 3-methyl-2-butenyl ester (basic prenyl ester)	40.68
111	Caffeic acid 3-methyl-3-butenyl ester (prenyl ester isomer I)	41.16
112	Dihydrosakuranetin chalcone	41.56
113	Unidentified	41.66
114	2-Acetyl-1-caffeoyl-3-*p*-coumaroylglycerol	41.79
115	Chrysin	42.12
116	Caffeic acid benzyl ester	42.55
117	2-Acetyl-3-caffeoyl-2-feruloylglycerol	42.59
118	Flavonoid	42.60
119	Unidentified	42.71
120	Pinocembrin (Dihydrochrysin)	43.07
121	Pinocembrin chalcone	43.28
122	Naringenin 7-methyl ether (Sakuranetin)	43.32
123	Unidentified	43.88
124	Naringenin 4'-methyl ether (Isosakuranetin)	44.31
125	Galangin	44.82
126	Isosakuranetin dihydrochalcone	44.91
127	Pinocembrin dihydrochalcone	45.45
128	Apigenin 4'-methyl ether (Acacetin) or Apigenin 7-methyl ether (Genkwanin)	45.45
129	Caffeic acid pentyl or isopentyl ester	46.52
130	Caffeic acid phenethyl ester (CAPE)	46.82
131	Kaempferol 4'-methyl ether (Kaempferide)	46.89
132	Pinobanksin 3-acetate	47.27
133	Kaempferol methyl ether isomer II	47.52
134	Tetramethyl flavonoid	47.84
135	Methoxychrysin	47.87
136	2',6'-Dihydroxy-4'-methoxypentanophenone	48.00
137	Unidentified	48.12
138	Unidentified	48.93
139	Kaempferol-3,4′-dimethyeter (Ermanin)	49.93
140	*p*-Coumaric acid 3-methyl-3-butenyl ester	50.27
141	2-Acetyl-1,3-di-*p*-coumaroylglycerol	50.51
142	1-Acetyl-2-*p*-coumaroyl-3-feruloylglycerol	51.48
143	1-Acetyl-2,3-di-*p*-coumaroylglycerol	51.68
144	*p*-Coumaric acid 3-methyl-2-butenyl or 2-methyl-2-butenyl	51.75
145	*p*-Coumaric acid 3-methyl-2-butenyl or 2-methyl-2-butenyl	52.43
146	Unidentified	53.16
147	*p*-Coumaric acid benzyl ester	53.48
148	Unidentified	54.60
149	Ferulic acid benzyl ester	54.92
150	Caffeic acid phenylpropenyl ester	55.71
151	Caffeic acid phenylpropyl ester	55.85
152	Pinobanksin 3-propanoate	57.82
153	Caffeic acid hexyl or isohexyl ester isomer I	57.99
154	*p*-Coumaric acid phenethyl ester	58.08
155	Unidentified	58.78
156	Caffeic acid hexyl or isohexyl ester isomer II	59.51
157	Unidentified	59.68
158	Pinostrobin chalcone	60.28
159	Unidentified	61.08
160	2′,6′-Dihydroxy-4′,4-dimethoxydihydrochalcone (Calomelanone)	61.32
161	2',6'-Dihydroxy-4'-methoxydihydrochalcone	61.90
162	Tectochrysin	62.70
163	Pinobanksin 3-butenoate or isobutenoate	62.91
164	Pinocembrin 7-methyl ether (Pinostrobin)	63.20
165	Unidentified	63.92
166	*p*-Coumaric acid cinnamyl ester	63.94
167	Unidentified	64.26
168	Caffeic acid heptyl or isoheptyl ester	64.41
169	Pinobanksin 3-butanoate or isobutanoate	64.74
170	Unidentified	64.81
171	Balsacone A/B/E/F isomer I	65.06
172	Unidentified	65.21
173	Balsacone A/B/E/F isomer II	65.37
174	Unidentified	65.39
175	Pinobanksin 3-pentenoate or isopentenoate isomer I	65.40
176	Balsacone C or Balsacone D	65.72
177	Unidentified	65.74
178	Pinobanksin 3-pentenoate or isopentenoate isomer II	65.74
179	Unidentified	65.76
180	Unidentified	66.01
181	Unidentified	66.10
182	Unidentified	66.24
183	Unidentified	66.57
184	Unidentified	66.60
185	Pinobanksin 3-benzoate	66.80
186	Unidentified	67.12
187	Pinobanksin derivate	67.49
188	Unidentified	67.63
189	Pinobanksin 3-pentanoate or isopentanoate isomer I	67.76
190	Pinobanksin 3-pentanoate or isopentanoate isomer II	67.91
191	Caffeic acid monoterpene (geranyl) ester	68.07
192	Unidentified	68.20
193	Unidentified	68.49
194	Pinobanksin 3-hexenoate or isohexenoate	68.54
195	Unidentified	68.70
196	Ricinoleic acid or 8-(3-octyloxiran-2-yl)octanoic acid	68.84
197	Balsacone L	68.86
198	Pinobanksin 3-cinnamate	68.89
199	Unidentified	69.01
200	Pinobanksin 3-hydroxycinnamate	69.16
201	Balsacone J or Balsacone P	69.16
202	Metoxycinnamic acid cinnamyl ester	69.27
203	Unidentified	69.29
204	Pinobanksin 3-hexanoate or -isohexanoate isomer I	69.57
205	Pinobanksin 3-heptenoate or -isoheptenoate isomer I	69.72
206	Metoxycinnamic acid cinnamyl ester isomer II	69.82
207	Pinobanksin 3-hexanoate or -isohexanoate isomer II	69.87
208	Iryantherin D or Balsacone K	70.20
209	Unidentified	70.26
210	Unidentified	70.64
211	Unidentified	70.78
212	Pinobanksin 3-phenylpentenoate or phenylisopentenoate ester	70.97
213	Unidentified	71.37
214	2-Hydroxyethyl palmitate or 12-Hydroxystearic acid	71.52
215	Unidentified	71.84
216	Unidentified	72.43
217	Unidentified	72.48
218	Unidentified	73.07
219	Unidentified	73.85
220	Unidentified	74.37
221	Unidentified	74.70
222	Unidentified	76.33
223	Unidentified	77.88

TYPE	MIXED										HYDROXYCINNAMIC ACIDS GLYCERIDES						HYDROXYCINNAMIC ACIDS MONOESTERS				FLAVONOID + HYDROXYCINNAMIC ACID MONOESTERS																						FLAVONOID											
No.	P.M×P.TRI ETOH	P.M×P.TRI W/E	P.RO ETOH	P.RO W/E	P.TA.1×P.TRI ETOH	P.TA.1×P.TRI W/E	P.TA.2×P.TRI ETOH	P.TA.2×P.TRI W/E	P.TRI ETOH	P.TRI W/E	P.LAS ETOH	P.LAS W/E	P.WIL ETOH	P.WIL. W/E	P.×WCA ETOH	P.×WCA W/E	P.N.1 ETOH	P.N.1 W/E	P.N.2 ETOH	P.N.2 W/E	P.BA ETOH	P.BA W/E	P.CA ETOH	P.CA W/E	P.M×P.B ETOH	P.M×P.B W/E	P.×PE.1 ETOH	P.×PE.1 W/E	P.×PE.2 ETOH	P.×PE.2 W/E	P.×RA ETOH	P.×RA W/E	P.SU ETOH	P.SU W/E	P.SI ETOH	P.SI W/E	P.TA.1 ETOH	P.TA.1 W/E	P.TA.2 ETOH	P.TA.2 W/E	P.×KOM ETOH	P.×KOM W/E	P.DE ETOH	P.DE W/E	P.DE×P.N ETOH	P.DE×P.N W/E	P.×ER ETOH	P.×ER W/E	P.LAU ETOH	P.LAU W/E	P.MAX ETOH	P.MAX W/E	P.N.3 ETOH	P.N.3 W/E
1	-	-	-	-	-	-	-	-	-	-	-	-	+	-	tr	-	-	-	-	-	-	-	-	-	-	-	-	-	-	-	-	-	-	-	-	-	-	-	-	-	-	-	-	-	-	-	-	-	-	-	-	-	-	-
2	-	+	-	++	-	+	-	tr	-	++	-	++	-	+++	-	+++	-	+	-	tr	-	++	-	+	-	++	-	+++	-	+	-	++	-	+	-	+	-	+	-	++	-	+	-	-	-	-	-	+	-	tr	-	++	-	+
3	+	tr	tr	-	tr	tr	tr	tr	tr	-	tr	-	-	-	tr	-	+	tr	+	tr	tr	-	tr	+	tr	tr	tr	tr	tr	tr	tr	-	tr	tr	+	+	tr	-	tr	-	+	+	+	+	+	+	tr	-	tr	tr	tr	tr	tr	-
4	-	+	-	tr	-	+	-	tr	-	tr	-	+	-	+	-	+	-	tr	-	+	-	+	-	+	-	+	-	+	-	+	-	+	-	tr	-	+	-	+	-	+	-	+	-	tr	-	tr	-	tr	-	tr	-	+	-	tr
5	tr	tr	tr	tr	tr	tr	tr	tr	tr	tr	-	-	tr	tr	tr	tr	tr	tr	tr	tr	tr	tr	+	+	tr	tr	tr	tr	tr	tr	tr	tr	tr	tr	tr	tr	tr	tr	tr	tr	tr	tr	tr	tr	tr	tr	tr	tr	-	tr	tr	tr	tr	tr
6	-	-	-	-	-	-	-	-	-	-	tr	tr	tr	tr	tr	tr	tr	tr	tr	tr	-	-	tr	tr	-	-	-	-	-	tr	-	-	-	-	-	-	-	-	-	-	-	-	-	-	tr	tr	-	-	-	-	-	-	-	-
7	tr	tr	-	-	tr	tr	tr	tr	-	-	tr	tr	tr	tr	tr	tr	tr	tr	+	tr	tr	tr	tr	tr	-	tr	tr	tr	tr	tr	tr	tr	tr	tr	tr	tr	tr	tr	tr	tr	tr	tr	tr	tr	tr	tr	tr	-	tr	tr	tr	tr	tr	tr
8	tr	tr	-	-	-	tr	-	tr	-	-	tr	tr	tr	-	-	tr	tr	tr	tr	tr	tr	tr	tr	tr	-	-	-	-	tr	tr	tr	tr	tr	tr	tr	tr	tr	tr	tr	tr	tr	tr	tr	tr	tr	tr	-	-	-	-	-	-	tr	tr
9	-	-	-	-	tr	tr	-	-	-	-	-	-	-	-	-	-	tr	tr	tr	tr	tr	tr	tr	tr	tr	tr	tr	tr	tr	tr	tr	tr	tr	tr	tr	-	tr	tr	tr	tr	tr	tr	-	-	-	-	-	-	-	-	-	-	-	-
10	-	-	-	-	-	-	-	-	-	-	-	-	-	-	-	-	-	-	tr	tr	tr	tr	-	-	-	-	-	-	-	-	tr	tr	-	-	-	-	tr	tr	tr	tr	-	-	-	-	-	-	-	-	-	-	-	-	-	-
11	tr	tr	tr	tr	tr	tr	tr	tr	-	-	tr	tr	tr	tr	tr	tr	tr	tr	tr	tr	tr	tr	tr	tr	-	tr	tr	tr	tr	tr	tr	tr	tr	tr	tr	tr	tr	tr	tr	tr	tr	tr	tr	tr	tr	tr	tr	-	-	tr	tr	tr	tr	tr
12	tr	tr	-	-	tr	tr	tr	tr	-	-	+	tr	+	tr	tr	tr	tr	tr	tr	tr	-	-	-	-	-	-	-	-	tr	tr	-	-	tr	tr	tr	tr	-	-	-	-	-	-	tr	tr	tr	tr	-	-	-	-	-	-	tr	tr
13	tr	tr	-	-	tr	tr	tr	tr	-	-	+	tr	+	tr	tr	tr	tr	tr	tr	+	-	-	tr	tr	tr	tr	tr	tr	tr	tr	tr	tr	tr	tr	tr	tr	tr	tr	tr	tr	+	+	+	+	+	+	tr	-	tr	tr	tr	tr	tr	tr
14	tr	tr	++	+	tr	tr	-	tr	tr	tr	+	tr	tr	tr	tr	tr	+	tr	+	+	+	+	+	tr	+	+	+	+	++	++	+	+	tr	tr	+	+	+	+	+	++	+	+	tr	tr	tr	+	+	+	tr	tr	+	+	+	+
15	-	-	-	-	-	-	-	-	-	-	tr	tr	-	-	-	tr	-	-	-	-	-	-	-	-	-	-	-	-	-	-	-	-	-	-	-	-	-	-	-	-	-	-	-	-	-	-	-	-	-	-	-	-	-	-
16	-	-	-	-	-	-	-	-	-	-	tr	tr	tr	tr	tr	tr	tr	tr	tr	tr	-	-	-	-	-	-	-	-	tr	tr	-	-	-	-	-	-	-	-	-	-	-	tr	-	tr	-	-	-	-	-	-	-	-	tr	tr
17	tr	tr	-	-	+	+	++	++	tr	tr	-	-	-	-	-	-	-	-	-	-	-	-	-	-	-	-	-	-	-	-	-	-	-	-	-	-	-	-	-	-	-	-	-	-	-	-	-	-	-	-	-	-	-	-
18	-	-	-	-	-	-	-	-	-	-	tr	tr	tr	tr	-	tr	tr	tr	tr	tr	tr	tr	tr	tr	tr	tr	tr	tr	tr	tr	tr	tr	-	tr	tr	tr	tr	tr	tr	tr	-	-	-	-	-	-	-	-	-	-	tr	tr	-	-
19	tr	tr	-	-	-	-	-	-	-	-	-	-	-	-	-	-	-	-	tr	tr	-	-	-	-	-	-	-	-	-	-	-	-	-	-	-	-	-	-	-	-	-	-	-	-	tr	tr	-	-	-	-	-	-	-	tr
20	-	-	-	-	-	-	-	-	-	-	-	-	-	-	-	-	-	-	tr	tr	-	-	-	-	-	-	-	-	-	tr	-	-	-	-	-	-	-	-	-	-	-	tr	-	-	tr	tr	-	-	-	-	-	-	-	-
21	+++	+++	+++++	++++	++	++	++++	++++	++++++	+++++	tr	tr	tr	+	tr	tr	tr	tr	tr	tr	tr	tr	-	-	tr	tr	tr	tr	+	+	tr	tr	-	-	tr	tr	tr	tr	tr	tr	+	tr	+	+	+	+	tr	tr	-	tr	-	tr	++	++
22	-	-	-	-	-	-	-	-	-	-	tr	-	tr	+	-	-	-	-	-	-	-	-	-	-	-	-	-	-	-	-	-	-	-	-	-	-	-	-	-	-	-	-	-	-	-	-	-	-	-	-	-	-	-	-
23	tr	tr	tr	tr	tr	tr	tr	tr	tr	tr	-	-	tr	tr	tr	tr	tr	tr	tr	tr	tr	tr	+	tr	tr	tr	tr	tr	tr	tr	tr	tr	tr	tr	tr	tr	tr	tr	tr	tr	+	+	+	+	+	+	tr	tr	tr	tr	tr	tr	tr	tr
24	tr	tr	-	-	-	-	-	-	-	-	-	-	-	-	-	-	-	-	-	-	-	-	tr	tr	-	-	-	-	-	-	-	-	tr	tr	tr	tr	-	-	-	-	tr	tr	tr	tr	tr	tr	tr	-	-	tr	-	-	-	-
25	-	-	-	-	-	-	-	-	-	-	-	-	-	-	-	-	tr	-	-	-	-	-	-	-	-	-	-	-	-	tr	-	-	-	-	-	-	-	-	-	-	tr	tr	-	-	tr	tr	+	tr	-	-	-	-	tr	tr
26	-	-	-	-	-	-	-	-	-	-	-	-	-	-	-	-	tr	tr	tr	tr	-	-	-	-	-	-	tr	tr	-	-	-	-	-	-	tr	tr	-	-	-	-	-	-	-	-	-	-	-	-	-	-	-	-	-	-
27	-	-	-	-	-	-	-	-	-	-	-	-	-	-	-	-	tr	tr	tr	tr	tr	tr	-	-	-	-	tr	tr	tr	tr	tr	tr	-	-	-	-	tr	tr	tr	tr	tr	tr	-	-	-	-	-	-	-	-	-	-	tr	tr
28	-	-	-	-	tr	tr	-	-	-	-	-	-	-	-	-	-	tr	tr	tr	tr	tr	tr	tr	-	-	-	-	-	-	-	tr	tr	-	-	-	-	tr	tr	tr	tr	tr	tr	tr	tr	tr	tr	tr	-	-	-	-	-	tr	tr
29	tr	tr	-	-	-	-	tr	tr	-	-	-	-	-	-	-	-	-	-	-	-	tr	tr	tr	tr	-	tr	-	-	tr	tr	tr	tr	tr	tr	tr	tr	tr	tr	tr	tr	tr	tr	-	-	tr	tr	tr	tr	tr	tr	tr	tr	-	-
206	tr	tr	-	-	tr	tr	-	-	-	-	-	-	+	-	tr	-	+	tr	tr	tr	tr	-	-	-	-	-	tr	tr	tr	-	tr	-	-	-	-	-	tr	tr	tr	-	tr	tr	-	-	tr	tr	+	tr	-	-	-	-	tr	tr
207	-	-	-	-	tr	tr	-	-	-	-	-	-	-	-	-	-	-	-	-	-	-	-	-	-	-	-	-	-	-	-	-	-	-	-	-	-	-	-	-	-	+	+	++	++	tr	-	++	+	+	tr	+	tr	++	+
208	-	-	tr	-	tr	tr	tr	tr	tr	-	-	-	-	-	-	-	-	-	-	-	-	-	-	-	-	-	-	-	-	-	-	-	-	-	-	-	-	-	-	-	-	-	-	-	-	-	-	-	-	-	-	-	-	-
209	-	-	-	-	-	-	-	-	-	-	-	-	-	-	-	-	-	-	-	-	tr	-	-	-	-	-	-	-	tr	-	tr	-	+	tr	-	-	-	-	-	-	tr	-	-	-	tr	tr	+	tr	tr	-	tr	tr	tr	tr
210	tr	tr	tr	tr	tr	-	-	-	tr	-	tr	-	+	tr	+	tr	+	tr	+	tr	tr	tr	-	-	-	-	tr	tr	tr	tr	tr	tr	tr	-	tr	-	tr	tr	tr	tr	tr	tr	-	-	tr	tr	+	tr	tr	tr	tr	tr	+	tr
211	-	-	-	-	-	-	-	-	-	-	-	-	++	+	++	tr	-	-	-	-	-	-	-	-	-	-	-	-	-	-	-	-	-	-	-	-	-	-	-	-	-	-	-	-	-	-	-	-	-	-	-	-	-	-
212	-	-	-	-	-	-	-	-	-	-	-	-	-	-	-	-	-	-	-	-	-	-	-	-	-	-	-	-	-	-	-	-	-	-	-	-	-	-	-	-	tr	tr	-	-	-	-	-	-	-	-	-	-	+	tr
213	-	-	-	-	tr	tr	-	-	-	-	-	-	-	-	-	-	-	-	-	-	tr	-	-	-	-	-	-	-	tr	tr	tr	-	+++	+	tr	-	tr	-	tr	-	+	tr	+	tr	tr	tr	+++	+	tr	tr	+++	+	++	+
214	-	-	-	-	-	-	-	-	-	-	-	-	tr	tr	+	-	-	-	-	-	-	-	-	-	-	-	-	-	-	-	-	-	-	-	-	-	-	-	-	-	-	-	-	-	-	-	-	-	-	-	-	-	-	-
215	tr	-	tr	-	-	-	-	-	-	-	-	-	-	-	-	-	-	-	-	-	-	-	-	-	-	-	-	-	-	-	-	-	++	++	+	tr	-	-	-	-	tr	tr	tr	tr	tr	tr	+	+	-	-	tr	tr	tr	tr
216	-	-	-	-	-	-	-	-	-	-	-	-	-	-	-	-	tr	tr	tr	-	-	-	-	-	-	-	tr	-	-	-	-	-	-	-	tr	-	-	-	-	-	tr	-	-	-	-	-	+	tr	-	-	-	-	tr	-
217	-	-	-	-	-	-	-	-	tr	-	-	-	-	-	-	-	-	-	-	-	tr	-	-	-	-	-	-	-	tr	-	tr	-	-	-	-	-	tr	-	tr	-	-	-	-	-	-	-	-	-	+	+	-	-	-	-
218	-	-	-	-	tr	tr	-	-	-	-	-	-	-	-	-	-	-	-	-	-	-	-	-	-	-	-	-	-	-	-	-	-	tr	-	tr	-	-	-	-	-	+	tr	-	-	tr	tr	+	tr	-	-	tr	-	+	tr
219	-	-	-	-	-	-	-	-	-	-	-	-	-	-	-	-	-	-	-	-	-	-	-	-	-	-	-	-	-	-	-	-	+	tr	tr	-	-	-	-	-	tr	-	-	-	tr	tr	+	tr	tr	-	tr	-	tr	tr
220	-	-	-	-	-	-	-	-	-	-	-	-	-	-	-	-	-	-	-	-	-	-	-	-	-	-	-	-	-	-	-	-	-	-	-	-	-	-	-	-	-	-	-	-	-	-	+	tr	-	-	+++	++	-	-
221	-	-	-	-	tr	-	-	-	-	-	-	-	-	-	-	-	-	-	-	-	-	-	-	-	-	-	-	-	tr	-	-	-	++++	+	+	tr	-	-	-	-	tr	tr	+	+	+	tr	+++	++	-	-	+++	+	+	tr
222	-	-	-	-	-	-	-	-	tr	-	-	-	-	-	-	-	tr	-	tr	-	+	-	tr	-	tr	-	+	tr	tr	-	+	-	tr	-	tr	-	+	-	+	-	-	-	-	-	-	-	tr	-	-	-	-	-	-	-
223	-	-	-	-	-	-	-	-	tr	-	-	-	-	-	-	-	-	-	-	-	-	-	-	-	-	-	-	-	-	-	-	-	+	-	-	-	-	-	-	-	-	-	-	-	-	-	+	-	-	-	++	tr	tr	-
Extraction yield [% of dry mass]	43.79%	32.04%	52.06%	40.64%	34.99%	25.86%	47.51%	37.82%	48.42%	37.75%	8.13%	9.67%	6.54%	7.73%	15.49%	11.87%	26.30%	19.62%	34.62%	27.18%	40.27%	41.45%	28.50%	19.09%	38.93%	25.44%	35.96%	31.09%	39.63%	31.73%	40.47%	26.79%	39.08%	26.29%	35.24%	27.55%	39.99%	29.63%	44.95%	33.28%	40.57%	31.20%	35.04%	29.98%	38.02%	20.18%	32.19%	29.84%	45.46%	21.80%	43.04%	35.56%	46.07%	33.46%

Table legend

Relativeabundance for MS evaluated components

tr - intensity <5*10^4	tr
+ - 5*10^4< intensity >1.5*10^5	+
++ - 1.5*10^5< intensity >3*10^5	++
+++ - 3*10^5< intensity >4.5*10^5	+++
++++ - 4.5*10^5< intensity>6.0*10^5	++++
+++++ - 6.0*10^5< intensity>7.5*10^5	+++++
++++++ - 7.5*10^5< intensity>1.0*10^6	++++++
+++++++ - 1.0*10^6< intensity	+++++++

Poplar taxons acronyms: P.BA—*P. balsamifera*; P.CA—*P. cathayana*; P.DE—*P. deltoides*; P.DE×P.N—*P. deltoides* × *P. nigra*; P.ER—*P.* 'Eridano'; P. ×KOM—*P.* × *komarowii*; P.LAU—*P. laurifolia*; P.LAS—*P. lasiocarpa*; P.MAX—*P. maximowiczii*; P.M×P.B—*P. maximowiczii* × *P. berolinensis*; P.M×P.TRI—*P. maximowiczii* × *P. trichocarpa*; P.N—*P. nigra* (samples 1-3); P.×PE—*P.* × *petrowskiana* (samples 1-2); P.×RA—*P.* × *rasumoskowiana*; P.RO—*P. trichocarpa* 'Rochester'; P.SI—*P. simonii*; P.SU—*P. suaveolens*; P.TA—*P. tacamahaca* (sample 1 and 2); P.TA×P.TRI—*P. tacamahaca* × *P. trichocarpa* (samples 1-2); P.TRI—*P. trichocarpa*, P.WIL—*P. wilsonii*, P.×WCA—*P.* × *wilsocarpa*

Relative abundance for only tectochrysin and pinostrobin

tr - height <1 mAU	tr
+ - 1< height >5 mAU	+
++ - 5< height >10 mAU	++
+++ - 10< height >15 mAU	+++
++++ - 15< height >20 mAU	++++
+++++ - 20< height >25 mAU	+++++
++++++ - 25< height mAU	++++++
